# The Effects of Chinese Herbal Medicines on the Quorum Sensing-Regulated Virulence in *Pseudomonas aeruginosa* PAO1

**DOI:** 10.3390/molecules23040972

**Published:** 2018-04-21

**Authors:** Yee Meng Chong, Kah Yan How, Wai Fong Yin, Kok Gan Chan

**Affiliations:** 1 Division of Genetics and Molecular Biology, ISB, Faculty of Science, University of Malaya, Kuala Lumpur 50603, Malaysia; cym_um@hotmail.com (Y.M.C.); yinwaifong@yahoo.com (W.F.Y.); 2 Vice Chancellor Office, Jiangsu University, Zhenjiang 212013, China; 3 Institute of Biological Sciences, Faculty of Science, University of Malaya, Kuala Lumpur 50603, Malaysia

**Keywords:** quorum sensing, natural products, *Pseudomonas aeruginosa* PAO1, *Poria cum Radix pini*, *Angelica dahurica*, *Rhizoma cibotii*, *Schizonepeta tenuifolia*

## Abstract

The quorum sensing (QS) system has been used by many opportunistic pathogenic bacteria to coordinate their virulence determinants in relation to cell-population density. As antibiotic-resistant bacteria are on the rise, interference with QS has been regarded as a novel way to control bacterial infections. As such, many plant-based natural products have been widely explored for their therapeutic roles. These natural products may contain anti-QS compounds that could block QS signals generation or transmission to combat QS pathogens. In this study, we report the anti-QS activities of four different Chinese herbal plant extracts: *Poria cum Radix pini*, *Angelica dahurica*, *Rhizoma cibotii* and *Schizonepeta tenuifolia*, on *Pseudomonas aeruginosa* PAO1. All the plants extracted using hexane, chloroform and methanol were tested and found to impair swarming motility and pyocyanin production in *P.* *aeruginosa* PAO1, particularly by *Poria cum Radix pini*. In addition, all the plant extracts also inhibited violacein production in *C.* *violaceum* CV026 up to 50% while bioluminescence activities were reduced in *lux*-based *E. coli* biosensors, pSB401 and pSB1075, up to about 57%. These anti-QS properties of the four medicinal plants are the first documentation that demonstrates a potential approach to attenuate pathogens’ virulence determinants.

## 1. Introduction

*Pseudomonas aeruginosa* PAO1 is a Gram-negative bacterium that is well known to cause various diseases, such as respiratory system infections, urinary tract infections, dermatitis, soft tissue infections, as well as bone and joints infections [1]. However, the excessive and discriminate use of antibiotics has led to the emergence of bacteria with resistance towards the drugs, causing a global threat for public health. Researchers are exploring different alternatives, and one of them is targeting the bacterial quorum sensing (QS) system, a mechanism commonly used by a variety of bacteria in coordinating their communal behavior [2,3].

*P. aeruginosa* PAO1 has two types of QS systems, LasI/R and RhlI/R, in which they produce autoinducers acylhomoserine lactone (AHL), known as *N*-(3-oxododecanoyl)-l-homoserine lactone (3OC12-HSL) and *N*-butyryl-l-homoserine lactone (C4-HSL), respectively [4]. *P. aeruginosa* PAO1 also possesses a quinolone-based signaling system (PQS), adding another level of control in the QS network, as it provides a link between the *las* and *rhl* systems [5]. In addition, the recent discovery of the fourth QS communication signal, named the integrated QS signaling system (IQS), revealed the complexity of the QS system in *P. aeruginosa* PAO1, in which it plays a vital role in modulation of bacteria pathogenesis by allowing the bacteria to regulate their QS system along with the environmental stress cues [6,7]. Previous studies have shown that *P. aeruginosa* has the ability to produce and secrete various virulence factors such as extracellular enzymes, haemolysins, toxins, secondary metabolites, pili and alginate [8]. These virulence factors are believed to be the main contributors for both acute and chronic infections in immunocompromised patients as they can generally damage both tissues and organs as well as interfere with the human immune system defense mechanism [9].

The QS system among bacteria is centralized on signal generator, signal receptor and autoinducer signaling molecules. Hence, plant-based natural products could be used to target the QS system in three different ways, either by interfering with the synthesis of autoinducers by AHL synthase, degrading the autoinducers or disrupting the binding of autoinducers to the signal receptor. One of the strategies is to screen for the presence of any compound(s) in the natural products that could prevent the synthesis of signaling molecules and/or decrease the expression of the *luxI*-encoded AHL synthase. For instance, if there is no AHL being produced, the bacteria would not be able to sense when a quorum is reached and thus the QS-controlled virulence genes would not be activated. To date, most of the studies on anti-QS compounds emphasized on the transcriptional regulator, in which the compounds could be used to inhibit the binding of signaling molecules to the cognate LuxR signal receptor through competitive inhibition [10,11,12]. Anti-QS compounds such as plant natural products are extensively studied as they are potential candidates in anti-virulence therapy to control bacterial infections. Anti-virulence therapy is a form of quorum quenching (QQ) in which the anti-QS compounds do not kill the bacteria but attenuate the pathogens and thus prevent them from attacking the hosts [13].

Chinese herbal medicines are believed to hold a rich treasure of therapeutic effects and have been used for thousands of years in China to promote health and as prescriptions for specific ailments. Different parts of the plants including leaves, stems, flowers, seeds as well as roots have been tested and have shown promise as potential active ingredients for new modern drugs [14,15]. Some of the plants’ food extracts as well as their active compounds have been found to target the bacterial QS system and this can help to minimize the formation of multi-drug resistant bacteria [2,10,16,17]. Research have shown that plant-derived sources such as malabaricone C (from *Myristica cinnamomea*) [18], *Melicope lunu-ankenda* [19] and *Phyllanthus amarus* [20] could inhibit QS responses in bacteria without affecting their growth. Other higher plants such as garlic, carrot, chamomile, and water lily, as well as an array of peppers, have been reported to exhibit anti-QS activity on bacteria [21]. The pea seedlings and root exudates, on the other hand, were found to inhibit pigment production, exochitinase activity and protease activity in *C. violaceum* [22] while buttonwood, graceful sandmat, bottlebrush, black olive, Florida clover ash and live oak have tested positive for QQ activities in both *C. violaceum* and *A. tumefaciens* biosensor strains [23]. Besides that, furanones produced by *Delisea pulchra* were found to attenuate the virulence activity in *S. liquefaciens*, *V. harveyi*, *E. carotovora* and *P. aeruginosa* significantly without affecting their growth [24,25]. 

In this study, we examined the anti-QS properties of four different herbal plants—*Poria cum Radix pini*, *Angelica dahurica*, *Rhizoma cibotii* and *Schizonepeta tenuifolia* extracts—that are commonly used against QS-regulated phenotypes in *P. aeruginosa* PAO1. The effect of the plant extracts on bioluminescence production by *Escherichia coli* [pSB401] and *E. coli* [pSB1075] were also assessed.

## 2. Results and Discussion

### 2.1. Bacterial Growth

The effect of the plant extracts on the growth of *C. violaceum* CV026, *P. aeruginosa* PAO1 and *E. coli lux*-based biosensors were observed over the course of 24 h and all of the extracts tested at 1 mg/mL did not cause any inhibition in the growth of the bacteria or biosensors used (Supplementary Figures S1–S4).

### 2.2. Screening for Anti-Quorum Sensing Activities

#### 2.2.1. Quantitative Analysis of Violacein Production in *C. violaceum* CV026

**Figure 1:**
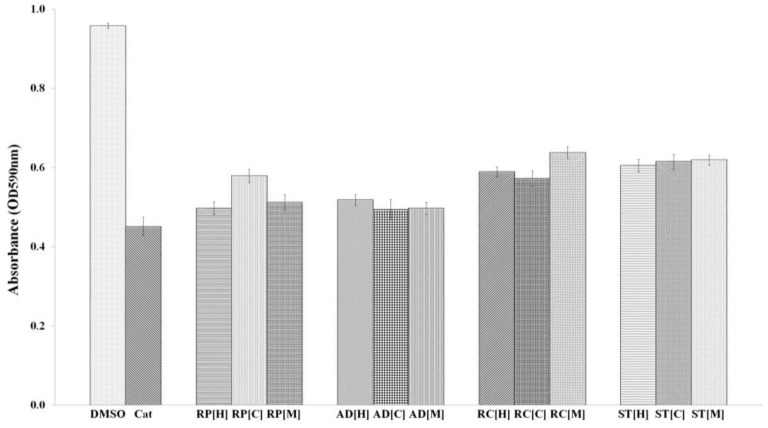
The inhibition of violacein production by the plant extracts, extracted using hexane [H], chloroform [C] and methanol [M]. *C. violaceum* CV026 incubated with DMSO and catechin (Cat) served as negative and positive controls, respectively. RP, AD, RC and ST correspond to plant extracts from *Poria cum Radix pini*, *Angelica dahurica*, *Rhizoma cibotii* and *Schizonepeta tenuifolia* at the concentration of 1 mg/mL, respectively. All the compounds with different extraction solvents were found to decrease the production of violacein compared to the negative control.

A preliminary screening was conducted on the crude extracts using *C. violaceum* CV026. *C. violaceum* is a Gram-negative bacterium that produces purple violacein pigment that acts as an antioxidant, which protects the bacteria membrane against oxidative stress and is a QS-mediated phenotype. In this study, we used *C. violaceum* CV026 as a biosensor to test the anti-QS effects of the plant extracts. *C. violaceum* CV026 is basically a double mini-*Tn5* mutant that has lost the ability to produce AHL, and its LuxR homologue receptor, known as CviR, binds only to short-chain AHLs, particularly C6-HSL. Our findings (Figure 1) showed that all the four plant extracts reduced violacein production in *C. violaceum* CV026 up to 50%. The extracts may inhibit the binding of AHL to transcriptional regulator, CviR, as *C. violaceum* CV026 possess a defective *cviI* synthase gene [26,27]. 

#### 2.2.2. Bioluminescence Assay

**Figure 2:**
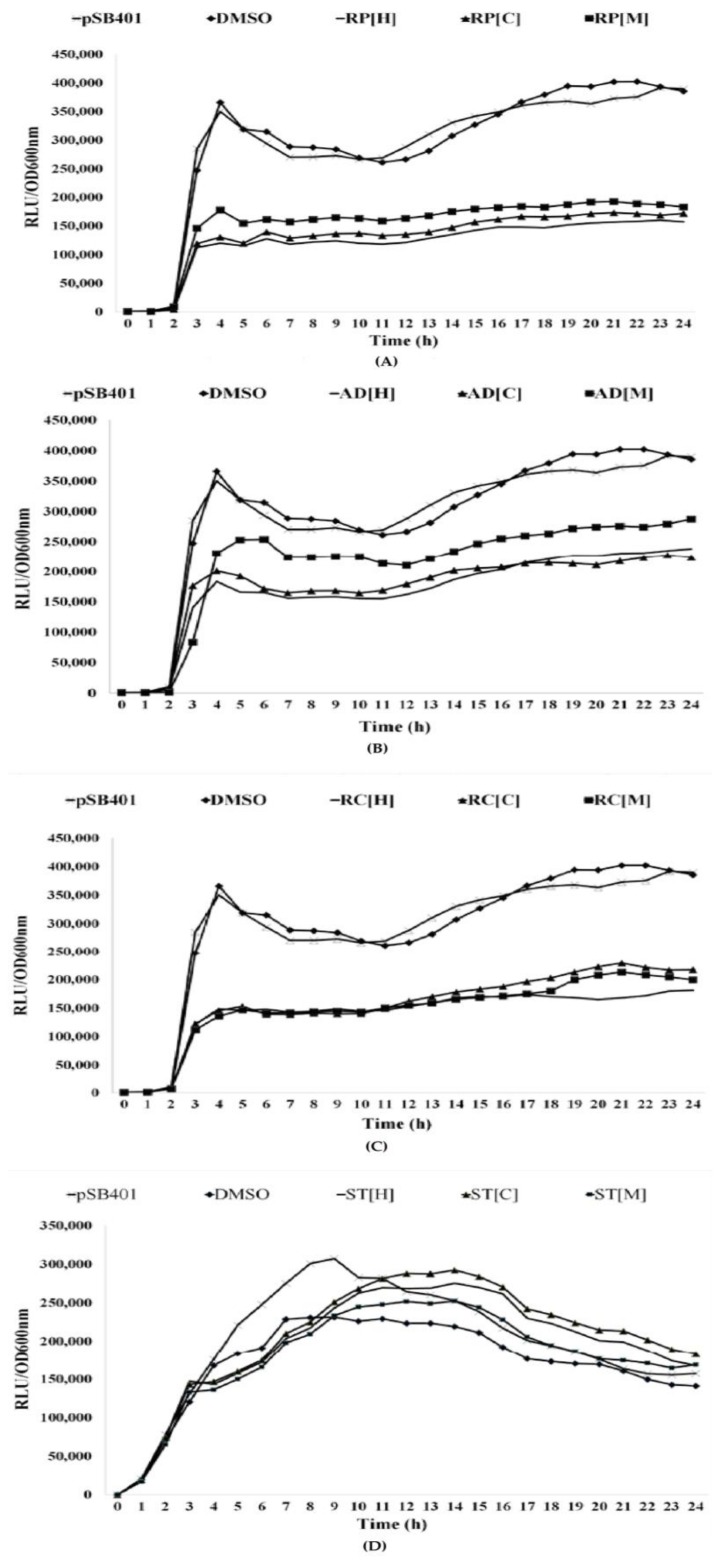
The inhibition of bioluminescence in *E. coli* pSB401 at the concentration of 1 mg/mL in hexane, chloroform and methanol. (**A**) *R. pini* (**B**) *A. dahurica* (**C**) *R. cibotii* (**D**) *S. tenuifolia*. The curve for pSB401 refers to untreated *E. coli* [pSB401] culture while DMSO served as negative control. Bioluminescence activities were found to decrease by half in all three of the plant samples, except for *A. dahurica* in methanol extracts. *S. tenuifolia*, on the other hand, shows negative results.

**Figure 3:**
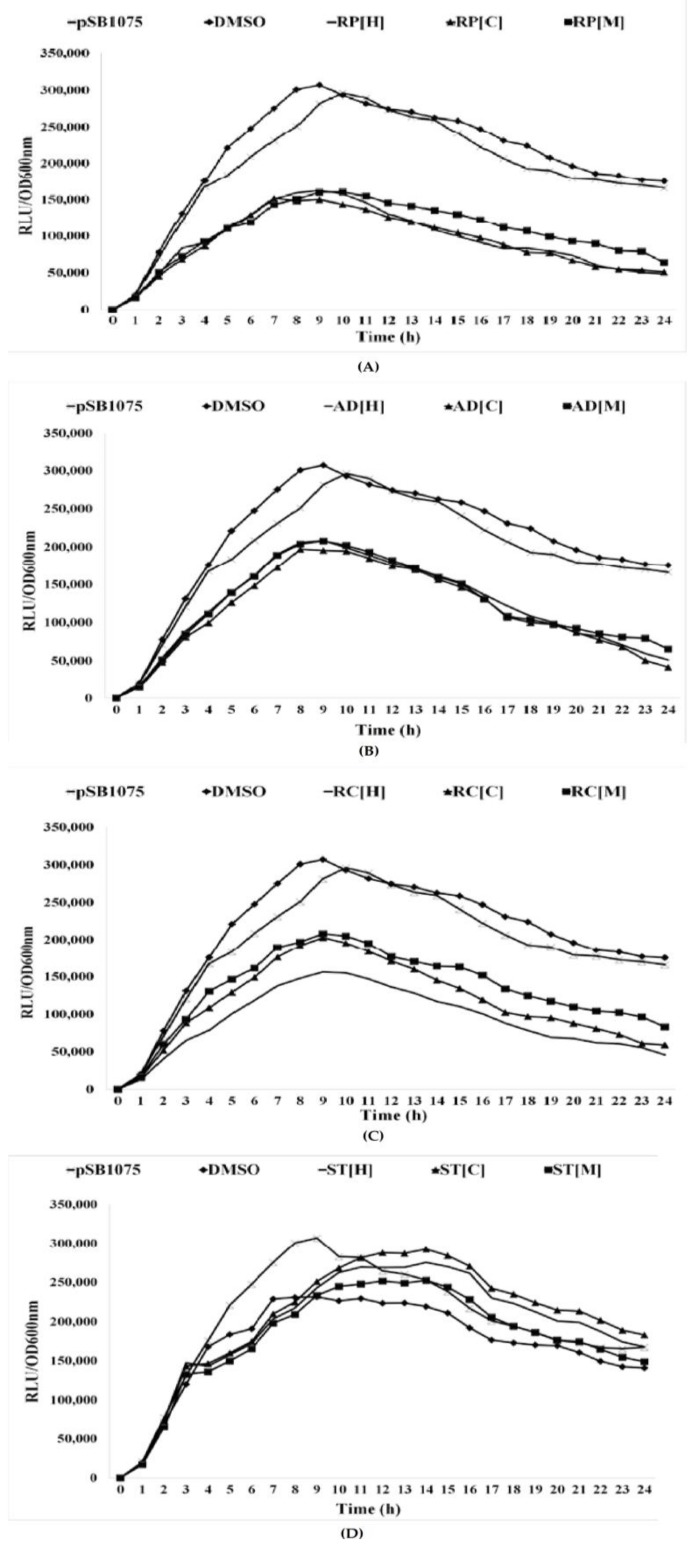
The inhibition of bioluminescence in *E. coli* [pSB1075] after being treated with 1 mg/mL of plant extracts in hexane, chloroform and methanol. (**A**) *R. pini* (**B**) *A. dahurica* (**C**) *R. cibotii* (**D**) *S. tenuifolia*, with DMSO served as negative control. All the plant samples show positive results in reducing bioluminescence production in *E. coli* [pSB1075], except for *S. tenuifolia*.

All the plant extracts were also tested for their ability to inhibit bioluminescence activities using *E. coli lux*-based biosensors. *E. coli* [pSB401] harbors *V. fischeri luxR* and the promoter region of *luxI* fused to *luxCDABE* from *Photorhabdus luminescens*. It responds to exogenous short-chain AHLs, resulting in light emission. Likewise, *E. coli* [pSB1075] carries *lasR* and promoter of *lasI* from *P. aeruginosa* PAO1 fused to *luxCDABE* of *P. luminescens*. This biosensor detects the presence of long-chain AHLs [28]. The present study shows that all the Chinese herbal plants, except *S. tenuifolia*, exhibited significant inhibition of bioluminescence in both biosensors, up to 57% (Figure 2 and Figure 3). This highly suggests that the three plant extracts have promising anti-QS activities.

#### 2.2.3. Pyocyanin Assay

**Figure 4:**
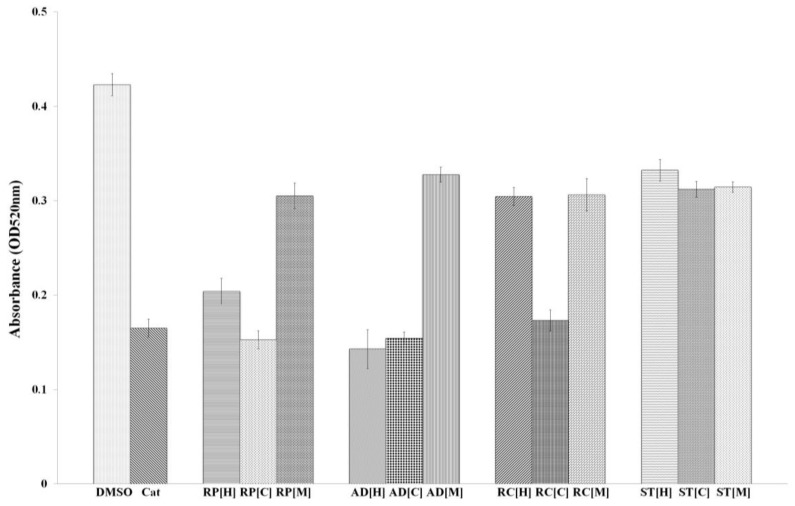
The inhibition of pyocyanin production by hexane [H], chloroform [C] and methanol [M]-extracted plant samples. *P. aeruginosa* PAO1 incubated with DMSO and catechin (Cat) served as negative and positive controls, respectively. RP, AD, RC and ST correspond to the plant extracts from *Poria cum R. pini*, *A. dahurica*, *R. cibotii* and *S. tenuifolia*, respectively, at the final concentration of 1 mg/mL. *A. dahurica* in hexane extract shows the strongest inhibition on the production of pyocyanin while *S. tenuifolia* shows the weakest effect among all the plant samples.

In recent years, extensive studies have been conducted on *P. aeruginosa* PAO1, an opportunistic human pathogen responsible for various infections in blood, skin, eye, gut, respiratory system, genitourinary tract and burn wounds. One of the major virulence determinants of *P. aeruginosa* PAO1 is pyocyanin, a blue secondary metabolite which could generate free-radicals. This is commonly seen in cystic fibrosis patients, in which pyocyanin interferes with ion transports as well as mucus secretion in the respiratory epithelial cells [29]. A number of studies have shown that the synthesis of pyocyanin is regulated by a complex synchrony of QS systems, *lasR-lasI*, *rhlR-rhlI* and *mvfR-haq* QS-system, in which they affect rhamnolipids, proteases and elastase production [30,31,32]. In this work, the anti-QS activity of the plants was validated by examining their effects on pyocyanin production in *P. aeruginosa* PAO1. Our results (Figure 4) showed that all the plant samples demonstrated an overall decrease in pyocyanin production when supplemented to the bacterial culture at a concentration of 1 mg/mL. The effect of *R. pini*, *A. dahurica* and *R. cibotii* on pyocyanin production suggests the presence of compounds which act as inhibitors of *rhl* system in the plant extracts [33]. However, it remains unknown if the compounds act on RhlI or RhlR, and further work needs to be done.

#### 2.2.4. Swarming Motility Assay

**Figure 5:**
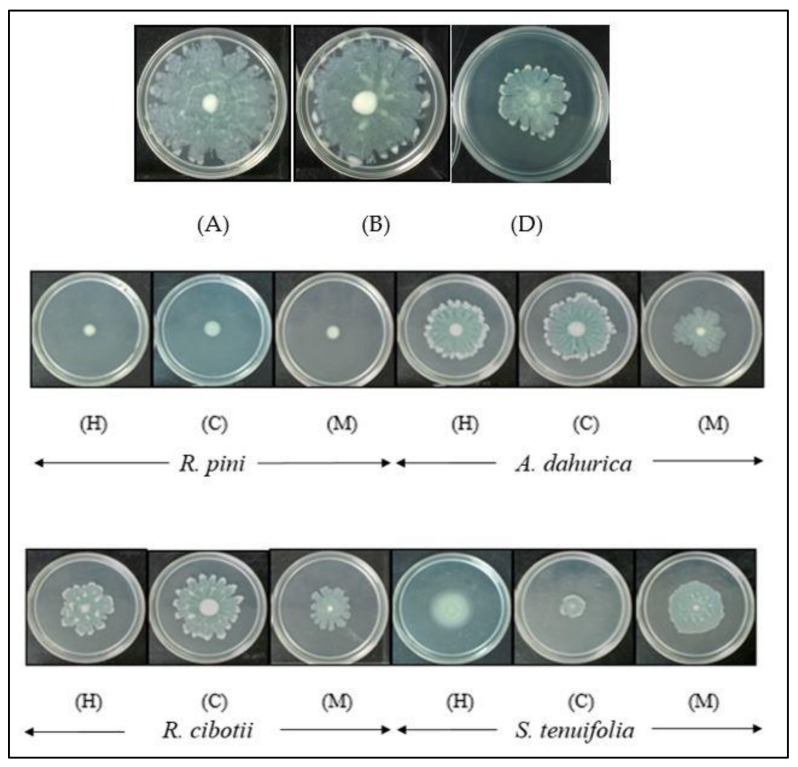
The effects of the four plant extracts (*R. pini*, *A. dahurica*, *R. cibotii* and *S. tenuifolia*) on the swarming motility in *P. aeruginosa* PAO1 at the concentration of 1 mg/mL in hexane [H], chloroform [C] and methanol [M] extraction. Swarming agar inoculated with (A) *P. aeruginosa* PAO1 alone, (B) *P. aeruginosa* PAO1 supplemented with DMSO (10% *v*/*v*), and (D) *P. aeruginosa* PAO1 supplemented with Malabaricone C at 1 mg/mL. All the samples reduce swarming in PAO1 with *R. pini* showing the strongest effect.

Another reason that *P. aeruginosa* PAO1 is considered a major life-threatening opportunistic pathogen is the ability to attach to surface area to form biofilms as well as to mobilize and colonize other environments through motility. Previous findings have shown that *P. aeruginosa* PAO1 is one of the exceptional bacteria that have three different types of motility, which are swimming, swarming and twitching [34]. Swarming can be referred to as part of the surface translocation that requires pili or flagella in order to coordinate their behavior across viscous environments like semi-solid agar surfaces. It is reported to be part of the QS-mediated phenotype and studies have proved that *lasI*/*lasR* mutants reduced and delayed swarming while *rhlI*/*rhlR* mutants inhibit the ability of the bacteria to swarm. In addition, the production of the biosurfactant or rhamnolipids also assist in swarming motility of *P. aeruginosa* PAO1 as it could reduce the surface tension that enables movement across the surface [34,35,36]. Swarming assays were performed in this work to explore the potential of the Chinese herbal plants in anti-QS activity. Results showed that all the plant extracts, particularly *R. pini*, were able to reduce swarming in *P. aeruginosa* PAO1 (Figure 5). A more detailed finding on swarming activities is shown in Supplementary Figure S5. 

### 2.3. The Quorum Sensing Inhibition of Other Plant Compounds

Numerous studies in the past decade have reported the capacity of plant extracts and phytochemicals to inhibit bacterial QS mechanism and thereby control the expression of their virulence attributes. Some of the favorable characteristics that make plant extracts ideal QS inhibitors include their high chemical stability, highly effective low molecular mass molecules and, most importantly, their being harmless for human health [10].

Most of the antagonists found in natural products mimic the AHL signals, thus attaching to the AHL receptor and thereby affecting the binding of the bacteria signaling molecules [13,16,22,37]. One example is the phenolic extract from the fruits of *Eugenia uniflora* [38] and *Eugenia brasiliensis* [39] which was found to inhibit the production of violacein in *C. violaceum.* Similarly, *Rubus rosaefolius* (wild strawberry) was able to impede the formation of biofilm, swarming motility and violacein production in *C. violaceum*, *A. hydrophila* and *S. marcescens* [40]. In addition, flavonoids extracted from *Centella asiatica* L. were reported to possess the ability to inhibit violacein production in *C. violaceum* as well as the production of pyocyanin, swarming motility, formation of biofilm, elastolytic and proteolytic activities in *P. aeruginosa* PAO1 [41]. Other flavonoid compounds such as naringenin, taxifolin, catechin and flavanes-3-ol were found to reduce the effect of pyocyanin and elastase production in *P. aeruginosa* PAO1 [42,43,44].

Paczkowski et al. [45], on the other hand, found that flavonoids, namely phloretin, chrysin and naringenin, could bind to the LasR and RhlR ligand-binding domain and significantly reduce their ability to bind to DNA encoding QS-regulated promoters These flavonoids inhibit QS through non-competitive mechanism and structure-activity relationship analysis indicated that the presence of two hydroxyl groups in the flavonoid backbone is needed for inhibition of LasR/RhlR receptors.

Another noteworthy plant compound is the *Punica granatum* (pomegranate) extract which demonstrated anti-QS activity on *C. violaceum* and inhibition of QS activity on pathogenic bacteria, *Yersinia enterocolitica* and *E. carotovora*. It was found that AHL production was reduced by pomegranate extract due to the degradation–transformation of AHLs [46,47].

All the research on roles of natural products as QS inhibitors serve as an advantage when it comes to the development of anti-virulence drugs, as this can help to minimize the possibility of the bacteria to become resistance mutants while preserving beneficial flora in the host [13,16,48]. Nevertheless, there is a concern about the resistance of pathogenic bacteria to anti-virulence therapy. There are a few in-vitro studies that suggested resistance to some anti-virulence molecules such as brominated furanone C-30 [49,50]. Besides this, the specificity of the anti-virulence compounds is also important so as to minimize the effect of native microbiota [51]. As such, more detailed studies are needed to address these issues on anti-QS natural compounds before they can be further assessed in clinical trials.

## 3. Materials and Methods

### 3.1. Bacteria Strains and Culture Conditions

The bacterial strains and plasmids used in this study are listed in Table 1. All the bacteria were grown in Luria-Bertani (LB) medium (Scharlab, Barcelona, Spain) at 37 °C with shaking at 220 rpm except for *Chromobacterium violaceum* CV026 which was cultured at 28 °C. *E. coli lux*-based AHL biosensors (pSB401 and pSB1075) were supplemented with 20 µg/mL of tetracycline for growth.

### 3.2. Plant Samples and Extraction of Crude Extracts

The plant samples that were used in this study are listed in Table 2. The samples were purchased from the local Chinese Medical Hall and were first washed with sterile distilled water followed by rinsing with 70% (*v*/*v*) ethanol before drying in the oven at 50 °C for three days. The dried samples were ground into fine powder form and were extracted sequentially using hexane [H], chloroform [C] and methanol [M]. The extracts were subsequently filtered through Whatman No. 1 filter paper and the solvent was removed using rotary evaporator (EYELA, Tokyo, Japan). The crude extracts were resuspended with 100% dimethyl sulfoxide (DMSO) at a final concentration of 10 mg/mL and were diluted to a working concentration of 1 mg/mL using ultrapure water prior to be used.

### 3.3. Bacterial Growth

To test for any bactericidal activity of the plant extracts, the growth profile of *C. violaceum* CV026, *P. aeruginosa* PAO1 and *E. coli lux*-based biosensors were studied using previously reported method with slight modifications [64]. Overnight bacteria cultures were diluted and measured using the Biochrom Libra S4 UV-visible spectrophotometer (Biochrom, Cambridge, UK) to OD_600nm_ of 0.1 before transfer 230 µL of diluted cultures into each well of 96-wells microtitre plate. Then, 20 µL of plant extract was added to each well. The bacteria were incubated at their optimum temperature and the optical density at OD_600nm_ was measured every 30 min for 24 h using Tecan luminometer (Infinite M200, Tecan, Männedorf, Switzerland). The measurement of optical density was taken in triplicate readings, in three independent experiments to get the mean value.

### 3.4. Screening for Anti-Quorum Sensing Activities

#### 3.4.1. Quantitative Analysis of Violacein Production by *C. violaceum* CV026

The quantitative analysis of violacein was performed based on the previously reported method with slight modifications [18]. An overnight culture of *C. violaceum* CV026 was adjusted to OD_600nm_ of 1.2 and was supplemented with 0.125 µg/mL of *N*-hexanoyl-l-homoserine lactone (C6-HSL). One hundred microlitre of CV026 culture was then transferred into the 96-wells microtitre plate followed by the addition of 10 µL of plant samples. The microplate was incubated for 16 h at 28 °C with agitation at 220 rpm. Next, the plate was dried at 60 °C until all the medium had evaporated and 100 µL of DMSO was then added to each well to dissolve the dried violacein pigment. The plate was subsequently incubated for another 2 h at 28 °C with shaking. The absorbance for each of the well was measured at OD_590nm_ by Tecan luminometer (Infinite M200, Tecan, Männedorf, Switzerland). The experiments were performed in triplicates, in three independent experiments and the mean value was calculated. DMSO (10% *v*/*v*) and catechin served as negative and positive controls.

#### 3.4.2. Bioluminescence Assay

The bioluminescence assay was performed using the procedures reported previously with some modifications [65]. *E. coli* [pSB401] and *E. coli* [pSB 1075] were grown overnight in LB medium at 37 °C. The bacterial culture was diluted to OD_600nm_ of 0.1, separately, and 230 µL of the diluted culture was added into a white 96-wells microtitre plate. Then, 20 µL of plant extracts were added to the wells containing bacterial culture. For *E. coli* [pSB401], 0.001 µg/mL of synthetic *N*-(3-oxohexanoyl)-l-homoserine lactone (3OC6-HSL) was added while 0.0125 µg/mL of *N*-(3-oxodecanoyl)-l-homoserine lactone (3OC10-HSL) was added into *E. coli* [pSB1075]. The luminescence and turbidity of the cultures were assessed every 30 min for 24 h with a Tecan luminometer (Infinite M200, Tecan, Männedorf, Switzerland) at OD_600nm_. A graph was plotted based on luminescence given in relative light units (RLU) per unit of turbidity (OD_600nm_). The measurements of optical density were taken in triplicate readings, in three independent experiments to obtain the mean value.

#### 3.4.3. Pyocyanin Assay

The pyocyanin assay was performed as previously described [18]. Briefly, an overnight culture of *P. aeruginosa* PAO1 was diluted to OD_600nm_ of 0.1. Next, 4.5 mL of the diluted culture was supplemented with 500 µL of plant extract and incubated at 37 °C for 24 h. The treated cell culture was extracted with 3 mL of chloroform and was then mixed with 1 mL of 0.2 M hydrochloric acid. Upon centrifugation, 200 μL of the organic layer was collected and transferred into a 96-wells microtitre plate. The absorbance reading at OD_520nm_ was taken using a Tecan luminometer (Infinite M200, Tecan, Männedorf, Switzerland). The experiments were performed in triplicates, in three independent experiments to get the mean value. DMSO (10% *v*/*v*) and catechin served as negative and positive controls, respectively.

#### 3.4.4. Swarming Motility Assay

The swarming agar plates were prepared based on the method modified as described previously [66]. It consists of Bacto agar (0.6% *w*/*v*), Bacto peptone (0.6% *w*/*v*), yeast extract (0.2% *w*/*v*) and glucose (0.5% *w*/*v*). Next, 1 mL of plant samples were mixed together with 30 mL of agar before pouring into the petri dishes. The plates were then allowed to air-dry for 30 min and 1 µL of the overnight culture of *P. aeruginosa* PAO1 with OD_600nm_ of 0.1 was point-inoculated at the center of the agar surface. The plates were incubated at 37 °C for 24 h. The experiments were performed in triplicates, in three independent experiments in order to get the mean value. DMSO (10% *v*/*v*) and catechin served as negative and positive controls, respectively.

### 3.5. Statistical Analysis

All the assays to screen for anti-QS activities were performed in triplicate, in three independent assays. The significance of the data was presented as mean ± standard deviation (SD) and analyzed using ANOVA test (*p* < 0.05) using GraphPad Prism software (GraphPad Software, California, CA, USA).

## 4. Conclusions

The Chinese herbal plants used in this study have been appreciated as traditional remedies for many illnesses since thousands of years ago. In this work, the plants tested have been shown to possess the ability to quench QS-regulated virulence expression such as bioluminescence activity, swarming motility, and the production of pyocyanin and violacein in bacteria tested without any bactericidal effect. The plant samples showed broad-spectrum effects as they can modulate the *P. aeruginosa* QS system at multiple levels mediated by different AHL molecules regardless of short or long chain residues [23,67]. It is postulated that the active compounds in the plant extracts may interfere with the LuxR receptor protein instead of the AHL synthase, as *C. violaceum* CV026, *E. coli* [pSB401] and *E. coli* [pSB1075] biosensors do not have *lux*-based synthase. In other words, the plant extracts may contain molecules that are structurally similar to autoinducers (or AHL), hence blocking the AHL-mediated QS. The ability of the active compounds in the four medicinal plants to inhibit QS-related virulence may provide the stepping stone and contribute to further research to discover new anti-microbial agents without the risk of developing antibiotic resistance. Nonetheless, there are more validations needed to answer the queries that surfaced from this study, including the mechanisms used by the plant compounds to target the bacterial QS system.

## Figures and Tables

**Table 1:** Bacterial strains and plasmids used in this study.

Strains or Plasmids	Relevant Genotype/Description	Reference/Source
*P. aeruginosa* PAO1	Wild type prototroph 21	[52]
*C. violaceum* CV026	Double mini-Tn*5* mutant derived from ATCC 31532 * Kan^R^, * Hg^R^, *cviI*::Tn*5 xylE*, plus spontaneous * Str^R^, AHL biosensor	[26]
*E. coli* (pSB401)	luxR/I (*Photobacterium fischeri* [ATCC 7744])::*luxCDABE* (*Photorhabdus luminescens* [ATCC 299999]) pACYC184-derived, * Tet^R^	[28]
*E. coli* (pSB1075)	lasR/I (*P. aeruginosa* PAO1)::*luxCDABE* (*Photorhabdus luminescens* [ATCC 299999]) fusion in * Tet^R^	[28]

* Kan^R^, Hg^R^, Str^R^, Tet^R^ refer to resistance to kanamycin, mercury, streptomycin and tetracycline respectively.

**Table 2:** Plants tested for anti-quorum sensing (QS) activities.

Plants	Ommon Name	Part of Plant Tested	Pharmacological/Medicinal Uses
*Poria cum Radix pini* [RP]	Poria spirit/‘Fu shen’	Sclerotia	Eliminate dampness, insomnia, promotes urination, strengthens spleen and stomach, sedative activity [53,54]
*Angelica dahurica* [AD]	Root of the holy ghost/Wild angelica/‘Bai zhi’	Roots	Antidote for acne, ulcer, rheumatism, headache, rhinitis, abdominal pain, hysteria, skin diseases, erythema, toothache, sinusitis [55,56,57]
*Rhizoma cibotii* [RC]	Chain fern rhizome/‘Gouji’	Rhizomes	Replenish liver and kidney, strengthen bones and muscles, treatment of chronic rheumatism, backache, leucorrhea spermatorrhea, hemiplegia, numbness [58,59,60]
*Schizonepeta tenuifolia* [ST]	‘Jing Jie’	Whole plant	Fever, anti-inflammatory, diaphoretic, analgesic, anti-febrile, anti-spasmodic, headache, allergic dermatitis, eczema, psoriasis [61,62,63]

## Supplementary Materials

The following are available online, Figure S1: The growth curve of *C. violaceum* CV026 at the concentration of 0.1, 0.5, 1, 2, 3, 4 and 5 mg/mL of plant extracts in hexane (**A**), chloroform (**B**) and methanol (**C**). Figure S2: The growth curve of *E. coli* [pSB401] at the concentration of 0.1, 0.5, 1, 2, 3, 4 and 5 mg/mL of plant extracts in hexane (**A**), chloroform (**B**) and methanol (**C**). Figure S3: The growth curve of *E. coli* [pSB1075] at the concentration of 0.1, 0.5, 1, 2, 3, 4 and 5 mg/mL of plant extracts in hexane (**A**), chloroform (**B**) and methanol (**C**). Figure S4: The growth curve of *P. aeruginosa* PA01 at the concentration of 0.1, 0.5, 1, 2, 3, 4 and 5 mg/mL of plant extracts in hexane (**A**), chloroform (**B**) and methanol (**C**). Figure S5: The effects of the four plant extracts (*R. pini*, *A. dahurica*, *R. cibotii* and *S. tenuifolia*) on the swarming motility in *P. aeruginosa* PAO1 at the concentration of 1 mg/mL in hexane [H], chloroform [C] and methanol [M] extraction.
