# Supplementary material for: The Effects of Chinese Herbal Medicines on the Quorum Sensing-Regulated Virulence in Pseudomonas aeruginosa PAO1
Source: Molecules. 2018 Apr 21;23(4):972. doi: 10.3390/molecules23040972 (PMC6017394; doi:10.3390/molecules23040972)
Supplement: Supplementary file 1 [file molecules-23-00972-s001.pdf]

Supplementary data

A. Bacterial Growth

(i) *C. violaceum* CV026

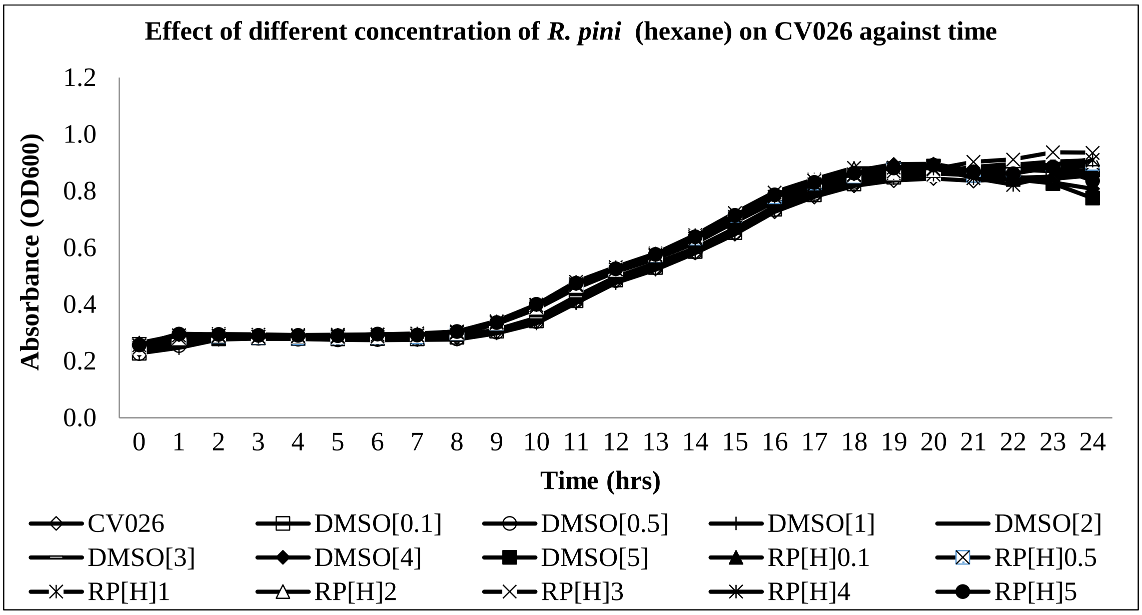

(1A)

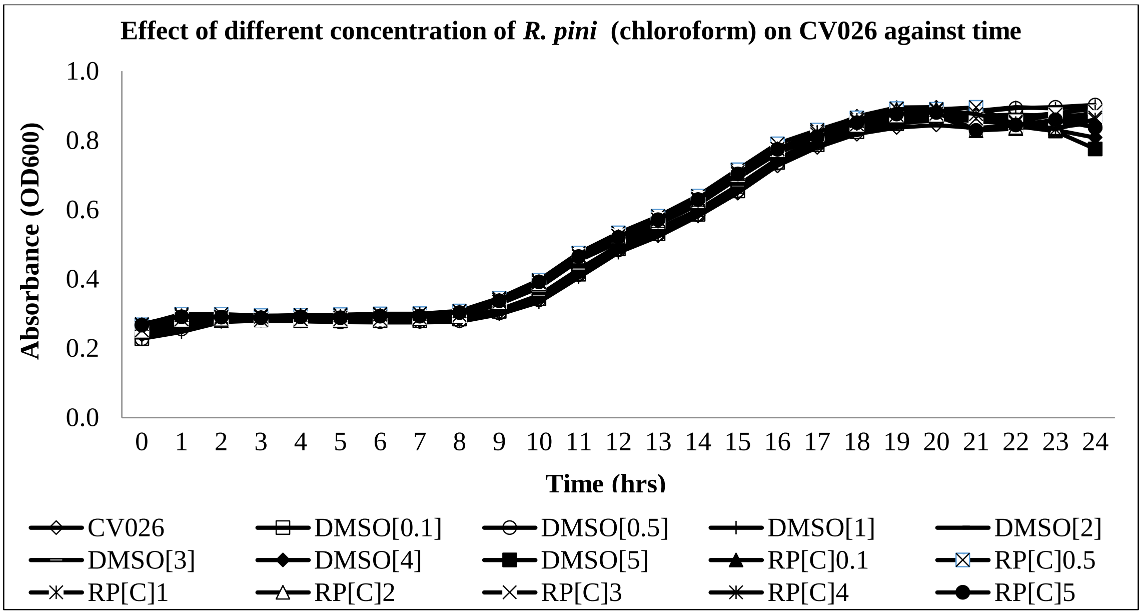

(1B)

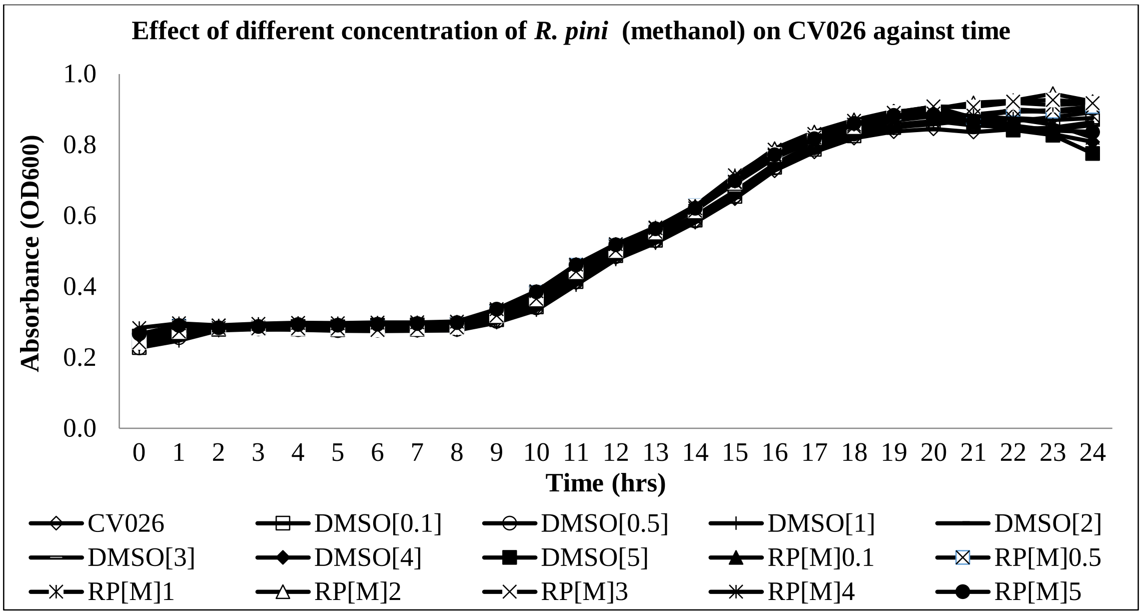
Effect of different concentration of R. pini (methanol) on CV026 against time
Absorbance (OD600)
1.0
0.8
0.6
0.4
0.2
0.0
0 1 2 3 4 5 6 7 8 9 10 11 12 13 14 15 16 17 18 19 20 21 22 23 24
Time (hrs)
CV026
DMSO[0.1]
DMSO[0.5]
DMSO[1]
DMSO[2]
DMSO[3]
DMSO[4]
DMSO[5]
RP[M]0.1
RP[M]0.5
RP[M]1
RP[M]2
RP[M]3
RP[M]4
RP[M]5

(1C)

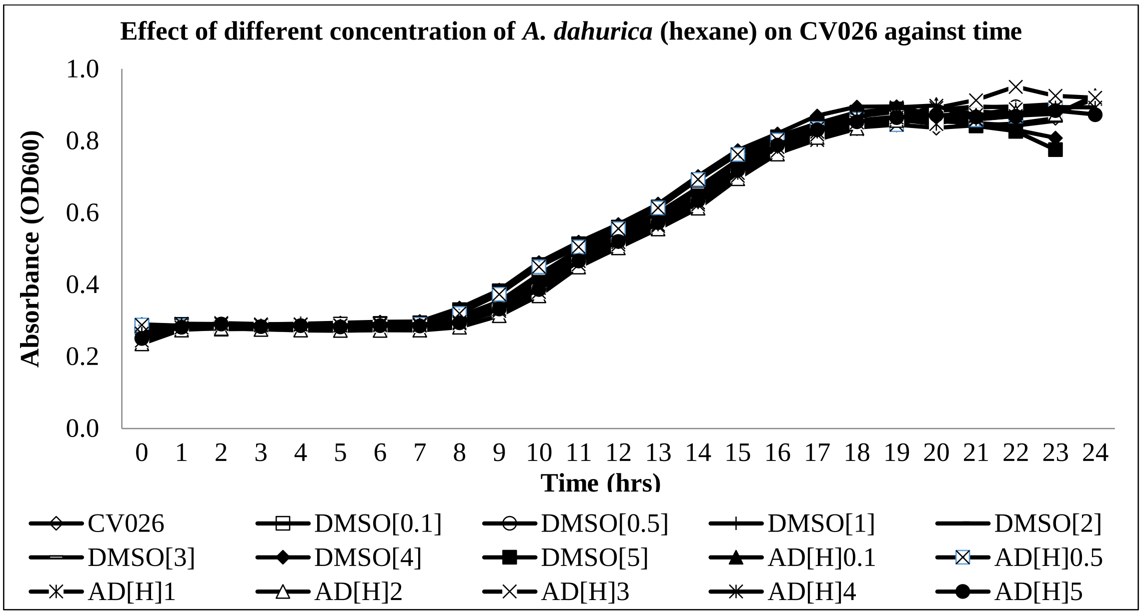
Effect of different concentration of A. dahurica (hexane) on CV026 against time
Absorbance (OD600)
1.0
0.8
0.6
0.4
0.2
0.0
0 1 2 3 4 5 6 7 8 9 10 11 12 13 14 15 16 17 18 19 20 21 22 23 24
Time (hrs)
CV026
DMSO[0.1]
DMSO[0.5]
DMSO[1]
DMSO[2]
DMSO[3]
DMSO[4]
DMSO[5]
AD[H]0.1
AD[H]0.5
AD[H]1
AD[H]2
AD[H]3
AD[H]4
AD[H]5

(2A)

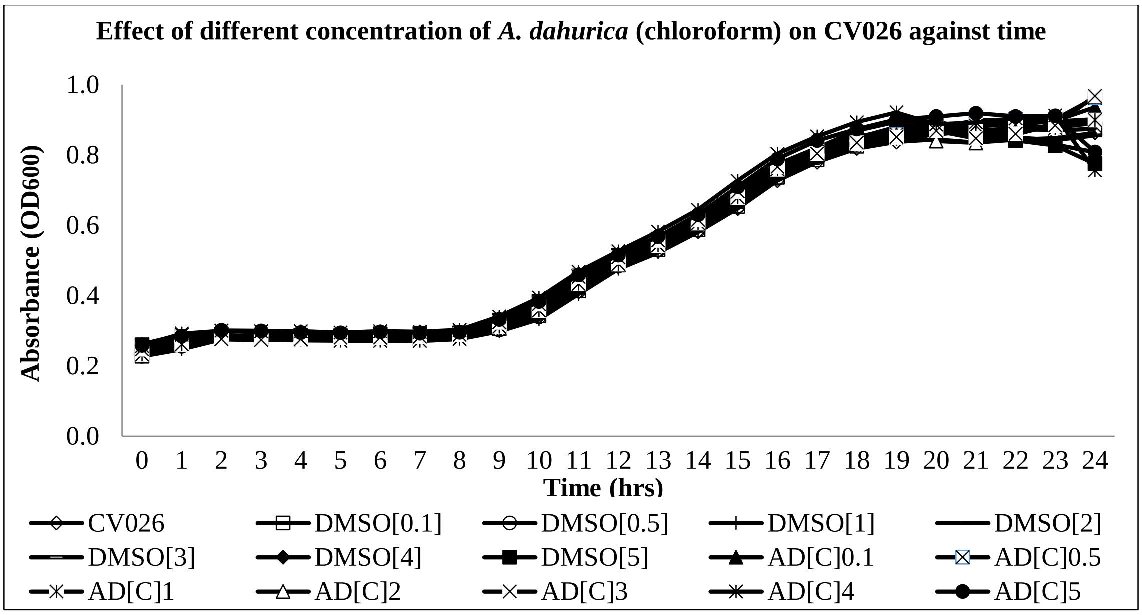
Effect of different concentration of A. dahurica (chloroform) on CV026 against time
Absorbance (OD600)
1.0
0.8
0.6
0.4
0.2
0.0
0 1 2 3 4 5 6 7 8 9 10 11 12 13 14 15 16 17 18 19 20 21 22 23 24
Time (hrs)
CV026
DMSO[0.1]
DMSO[0.5]
DMSO[1]
DMSO[2]
DMSO[3]
DMSO[4]
DMSO[5]
AD[C]0.1
AD[C]0.5
AD[C]1
AD[C]2
AD[C]3
AD[C]4
AD[C]5

(2B)

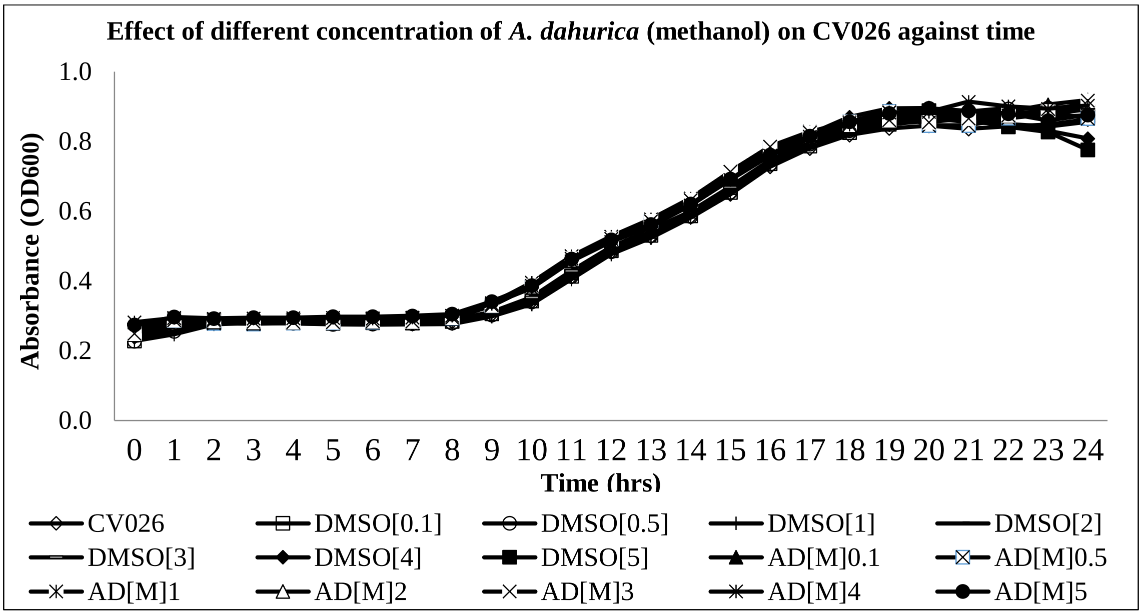
Effect of different concentration of A. dahurica (methanol) on CV026 against time
Absorbance (OD600)
1.0
0.8
0.6
0.4
0.2
0.0
0 1 2 3 4 5 6 7 8 9 10 11 12 13 14 15 16 17 18 19 20 21 22 23 24
Time (hrs)
CV026
DMSO[0.1]
DMSO[0.5]
DMSO[1]
DMSO[2]
DMSO[3]
DMSO[4]
DMSO[5]
AD[M]0.1
AD[M]0.5
AD[M]1
AD[M]2
AD[M]3
AD[M]4
AD[M]5

(2C)

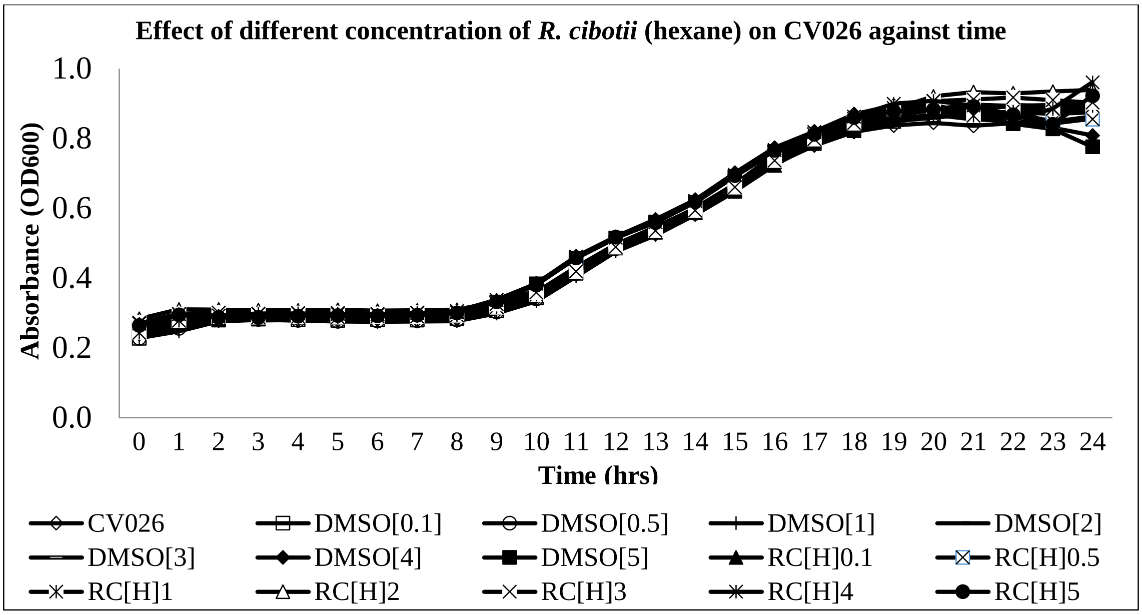
Effect of different concentration of R. cibotii (hexane) on CV026 against time
Absorbance (OD600)
1.0
0.8
0.6
0.4
0.2
0.0
0 1 2 3 4 5 6 7 8 9 10 11 12 13 14 15 16 17 18 19 20 21 22 23 24
Time (hrs)
CV026
DMSO[0.1]
DMSO[0.5]
DMSO[1]
DMSO[2]
DMSO[3]
DMSO[4]
DMSO[5]
RC[H]0.1
RC[H]0.5
RC[H]1
RC[H]2
RC[H]3
RC[H]4
RC[H]5

(3A)

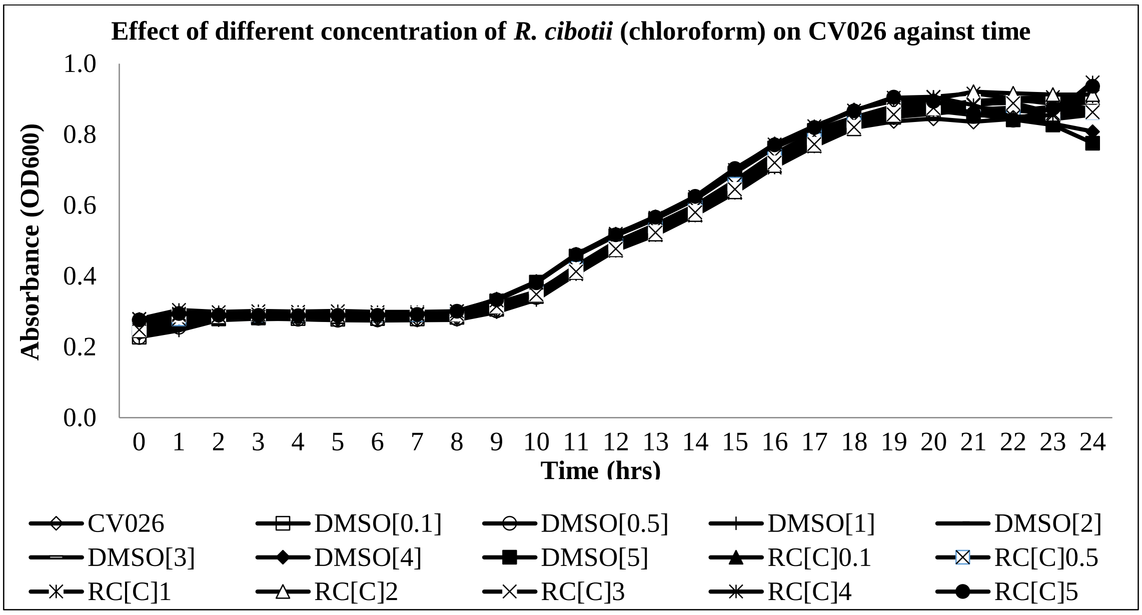
Effect of different concentration of R. cibotii (chloroform) on CV026 against time
Absorbance (OD600)
1.0
0.8
0.6
0.4
0.2
0.0
0 1 2 3 4 5 6 7 8 9 10 11 12 13 14 15 16 17 18 19 20 21 22 23 24
Time (hrs)
CV026
DMSO[0.1]
DMSO[0.5]
DMSO[1]
DMSO[2]
DMSO[3]
DMSO[4]
DMSO[5]
RC[C]0.1
RC[C]0.5
RC[C]1
RC[C]2
RC[C]3
RC[C]4
RC[C]5

(3B)

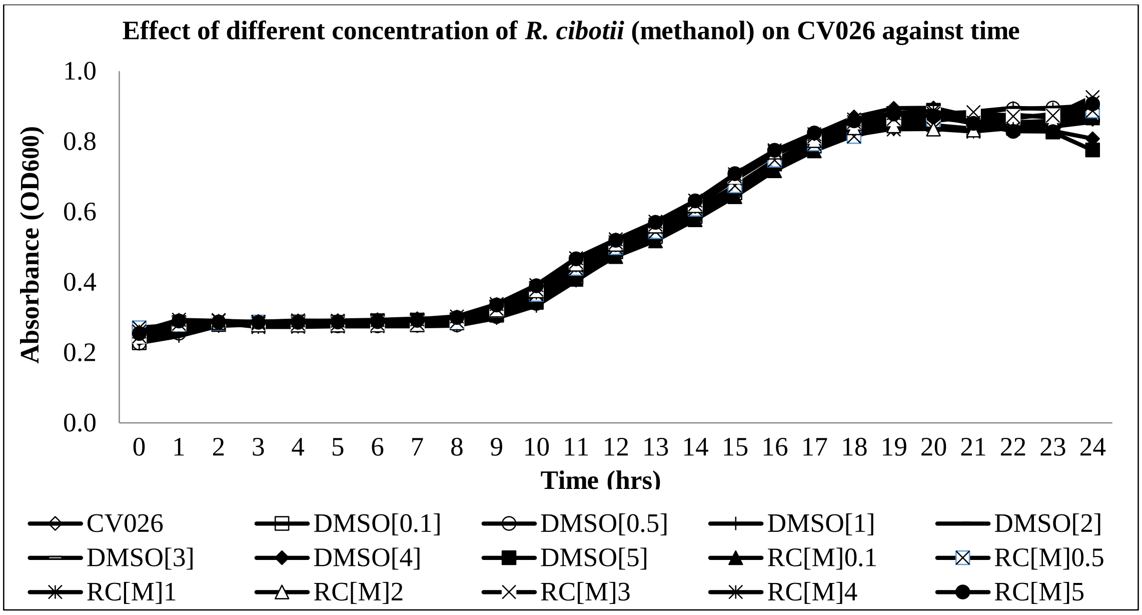
Effect of different concentration of R. cibotii (methanol) on CV026 against time
Absorbance (OD600)
1.0
0.8
0.6
0.4
0.2
0.0
0 1 2 3 4 5 6 7 8 9 10 11 12 13 14 15 16 17 18 19 20 21 22 23 24
Time (hrs)
CV026
DMSO[0.1]
DMSO[0.5]
DMSO[1]
DMSO[2]
DMSO[3]
DMSO[4]
DMSO[5]
RC[M]0.1
RC[M]0.5
RC[M]1
RC[M]2
RC[M]3
RC[M]4
RC[M]5

(3C)

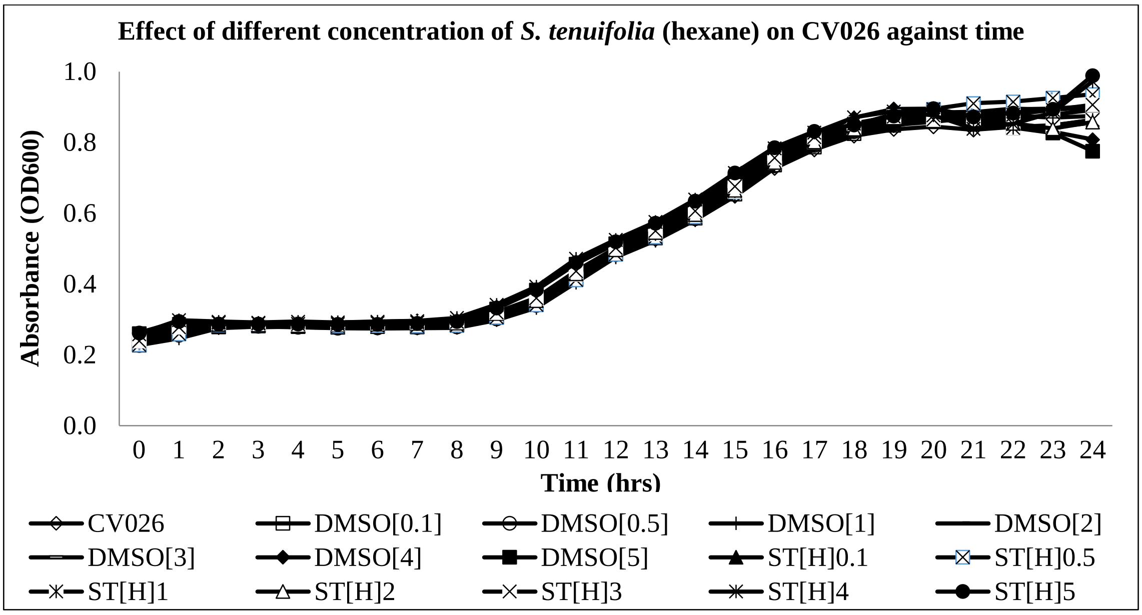
Effect of different concentration of S. tenuifolia (hexane) on CV026 against time
Absorbance (OD600)
1.0
0.8
0.6
0.4
0.2
0.0
0 1 2 3 4 5 6 7 8 9 10 11 12 13 14 15 16 17 18 19 20 21 22 23 24
Time (hrs)
CV026
DMSO[0.1]
DMSO[0.5]
DMSO[1]
DMSO[2]
DMSO[3]
DMSO[4]
DMSO[5]
ST[H]0.1
ST[H]0.5
ST[H]1
ST[H]2
ST[H]3
ST[H]4
ST[H]5

(4A)

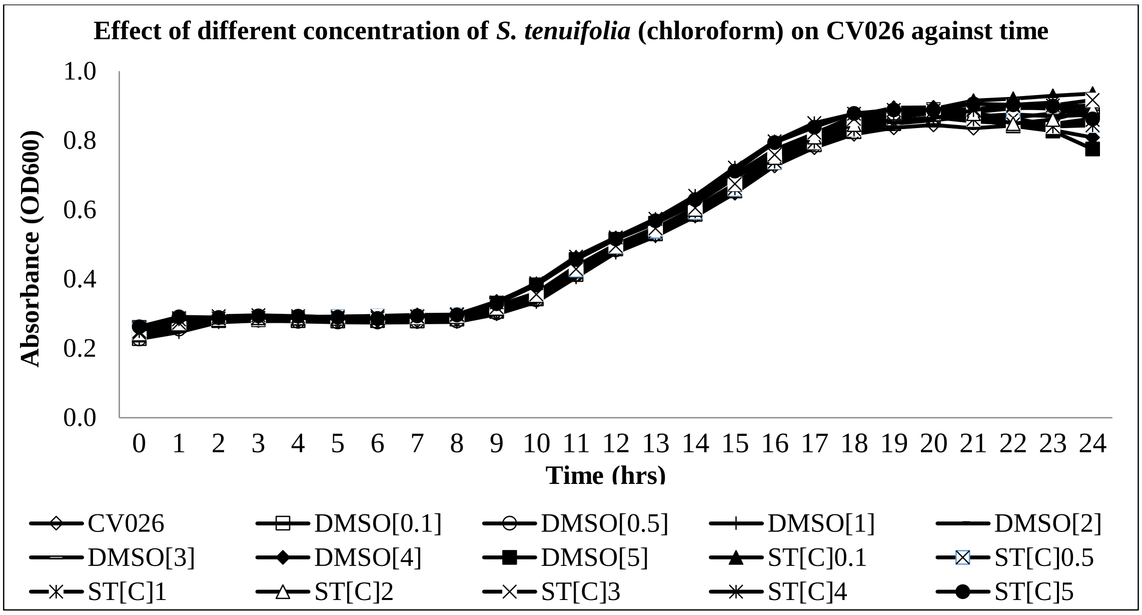
Effect of different concentration of S. tenuifolia (chloroform) on CV026 against time
Absorbance (OD600)
1.0
0.8
0.6
0.4
0.2
0.0
0 1 2 3 4 5 6 7 8 9 10 11 12 13 14 15 16 17 18 19 20 21 22 23 24
Time (hrs)
CV026
DMSO[0.1]
DMSO[0.5]
DMSO[1]
DMSO[2]
DMSO[3]
DMSO[4]
DMSO[5]
ST[C]0.1
ST[C]0.5
ST[C]1
ST[C]2
ST[C]3
ST[C]4
ST[C]5

(4B)

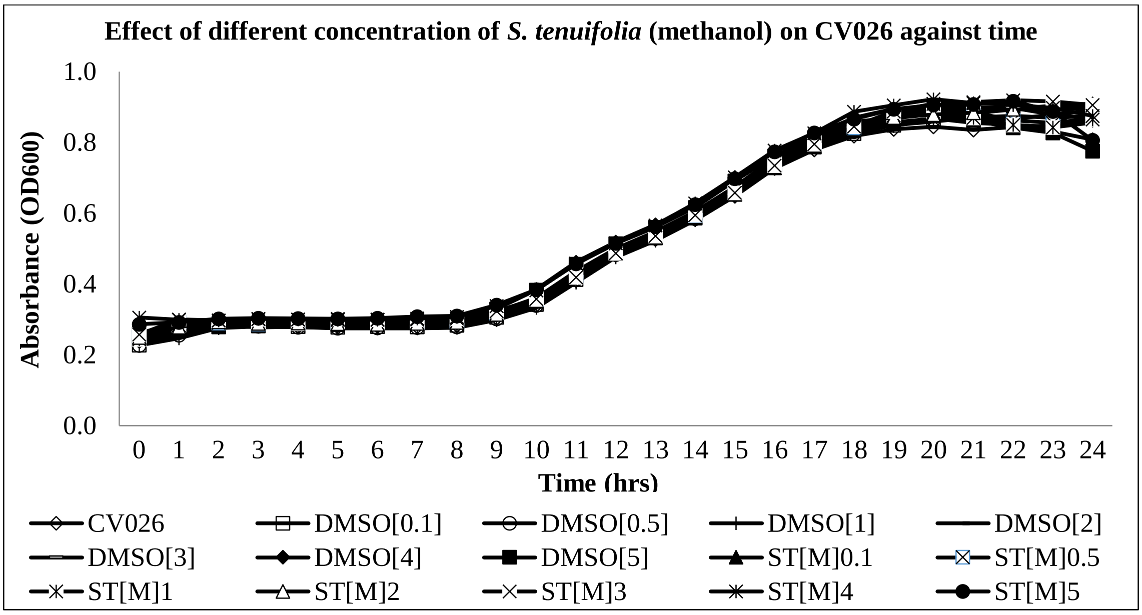
Effect of different concentration of S. tenuifolia (methanol) on CV026 against time
Absorbance (OD600)
1.0
0.8
0.6
0.4
0.2
0.0
0 1 2 3 4 5 6 7 8 9 10 11 12 13 14 15 16 17 18 19 20 21 22 23 24
Time (hrs)
CV026
DMSO[0.1]
DMSO[0.5]
DMSO[1]
DMSO[2]
DMSO[3]
DMSO[4]
DMSO[5]
ST[M]0.1
ST[M]0.5
ST[M]1
ST[M]2
ST[M]3
ST[M]4
ST[M]5

(4C)

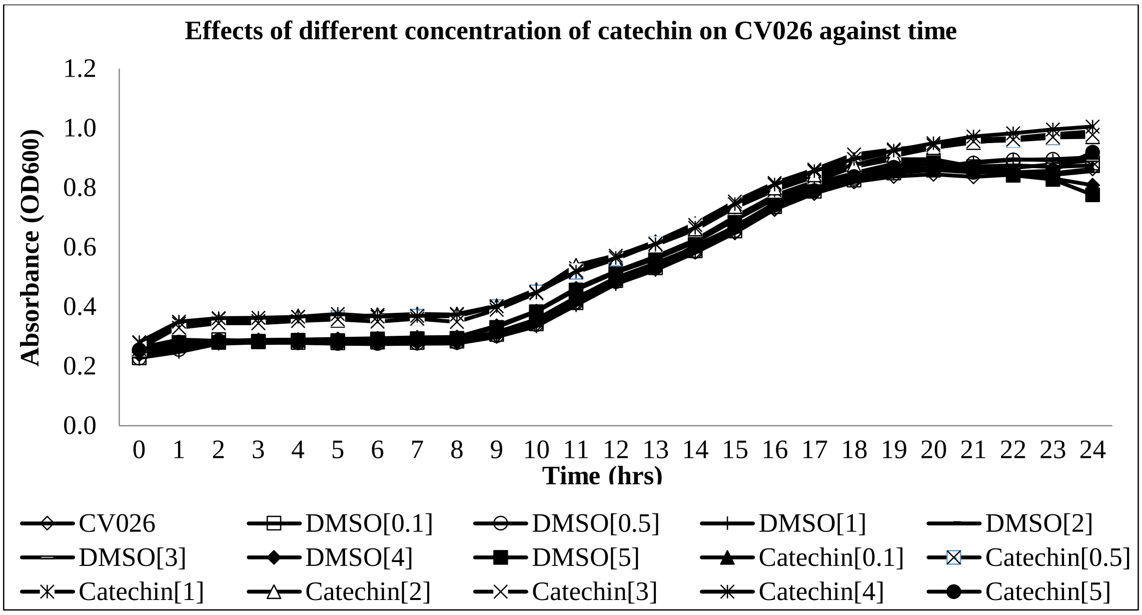

Supplementary Figure 1. The growth curve of *C. violaceum* CV026 at the concentration of 0.1, 0.5, 1, 2, 3, 4 and 5 mg/mL of plant extracts in hexane(A), chloroform (B) and methanol(C). (1) *R. pini* (2) *A. dahurica* (3) *R. cibotii* (4) *S. tenuifolia*. The curve for *C. violaceum* CV026 refers to untreated culture while DMSO and catechin served as negative and positive controls, respectively.

(ii) *E. coli* [pSB401]

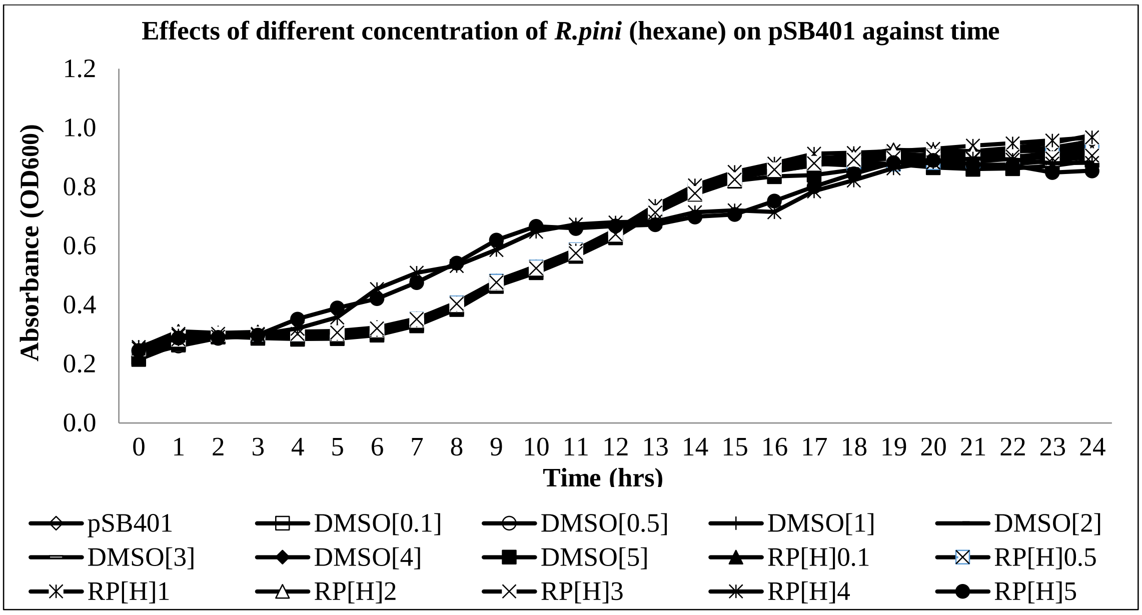

(1A)

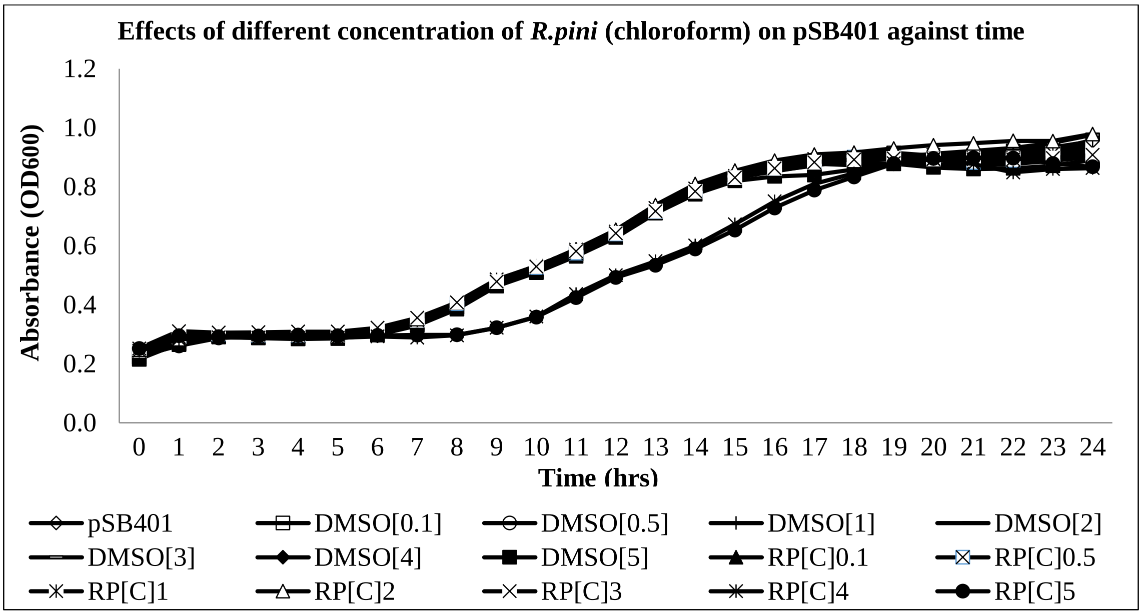

(1B)

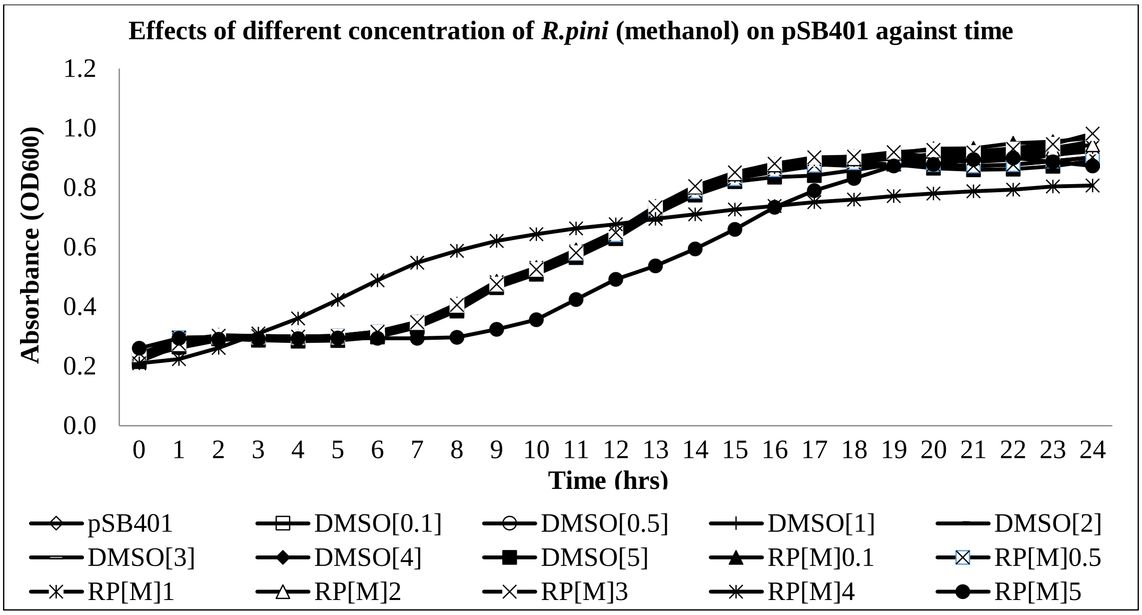

Effects of different concentration of R.pini (methanol) on pSB401 against time
Absorbance (OD600)
1.2
1.0
0.8
0.6
0.4
0.2
0.0
0 1 2 3 4 5 6 7 8 9 10 11 12 13 14 15 16 17 18 19 20 21 22 23 24
Time (hrs)
pSB401
DMSO[0.1]
DMSO[0.5]
DMSO[1]
DMSO[2]
DMSO[3]
DMSO[4]
DMSO[5]
RP[M]0.1
RP[M]0.5
RP[M]1
RP[M]2
RP[M]3
RP[M]4
RP[M]5

(1C)

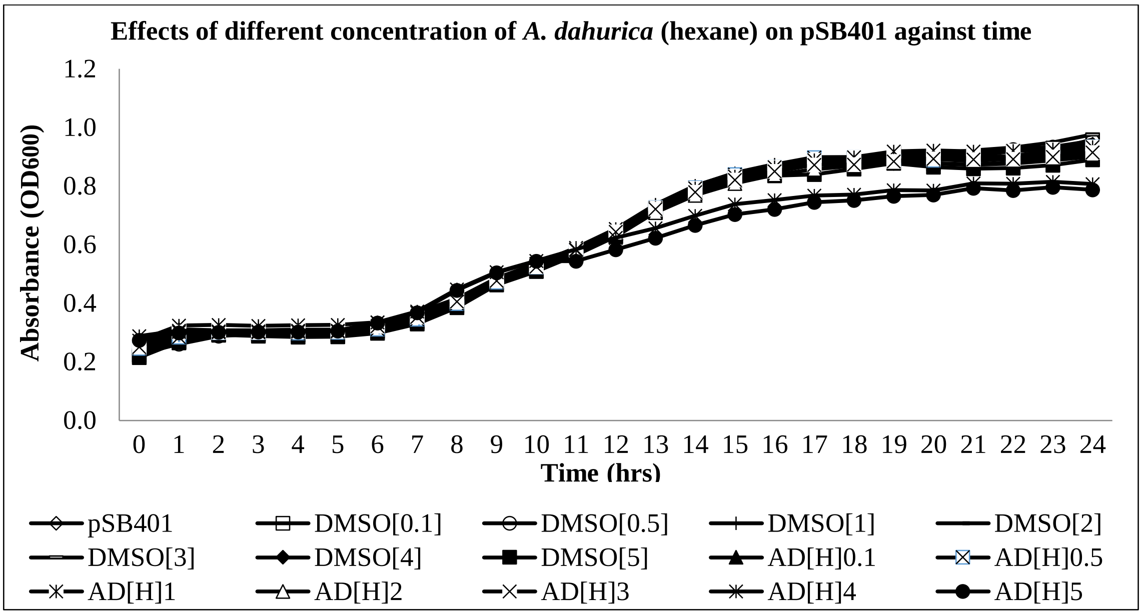

Effects of different concentration of A. dahurica (hexane) on pSB401 against time
Absorbance (OD600)
1.2
1.0
0.8
0.6
0.4
0.2
0.0
0 1 2 3 4 5 6 7 8 9 10 11 12 13 14 15 16 17 18 19 20 21 22 23 24
Time (hrs)
pSB401
DMSO[0.1]
DMSO[0.5]
DMSO[1]
DMSO[2]
DMSO[3]
DMSO[4]
DMSO[5]
AD[H]0.1
AD[H]0.5
AD[H]1
AD[H]2
AD[H]3
AD[H]4
AD[H]5

(2A)

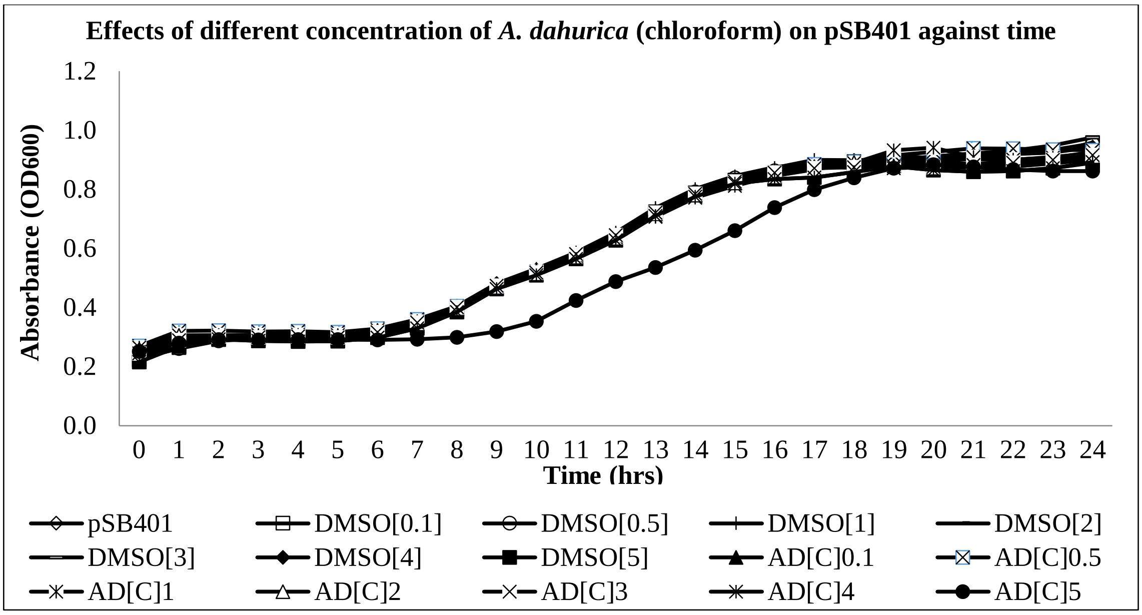
Effects of different concentration of A. dahurica (chloroform) on pSB401 against time
Absorbance (OD600)
1.2
1.0
0.8
0.6
0.4
0.2
0.0
0 1 2 3 4 5 6 7 8 9 10 11 12 13 14 15 16 17 18 19 20 21 22 23 24
Time (hrs)
pSB401
DMSO[0.1]
DMSO[0.5]
DMSO[1]
DMSO[2]
DMSO[3]
DMSO[4]
DMSO[5]
AD[C]0.1
AD[C]0.5
AD[C]1
AD[C]2
AD[C]3
AD[C]4
AD[C]5

(2B)

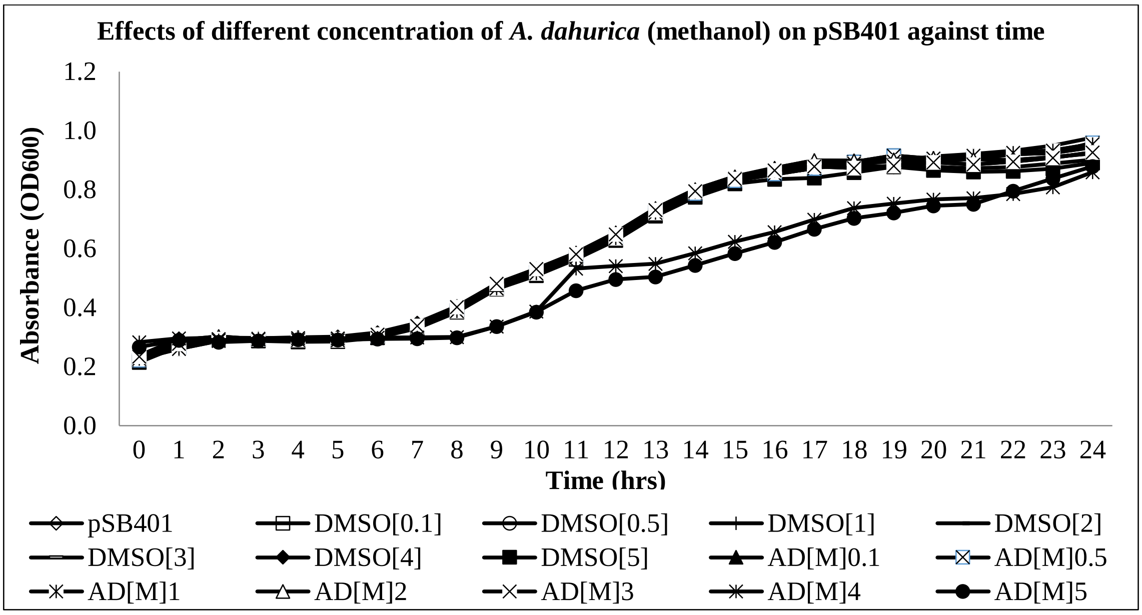
Effects of different concentration of A. dahurica (methanol) on pSB401 against time
Absorbance (OD600)
1.2
1.0
0.8
0.6
0.4
0.2
0.0
0 1 2 3 4 5 6 7 8 9 10 11 12 13 14 15 16 17 18 19 20 21 22 23 24
Time (hrs)
pSB401
DMSO[0.1]
DMSO[0.5]
DMSO[1]
DMSO[2]
DMSO[3]
DMSO[4]
DMSO[5]
AD[M]0.1
AD[M]0.5
AD[M]1
AD[M]2
AD[M]3
AD[M]4
AD[M]5

(2C)

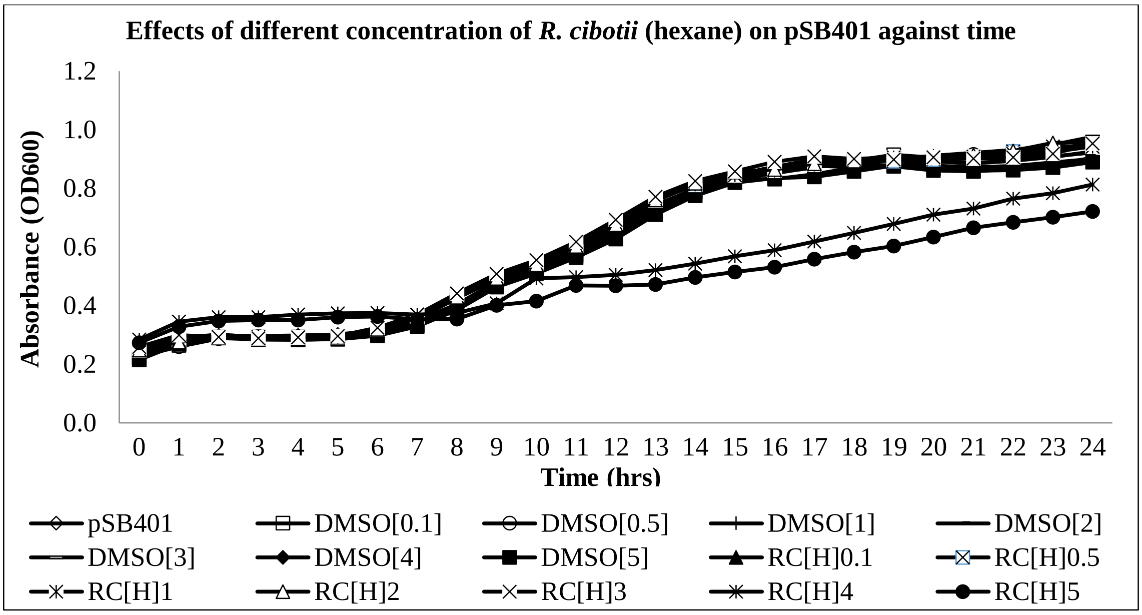
Effects of different concentration of R. cibotii (hexane) on pSB401 against time
Absorbance (OD600)
1.2
1.0
0.8
0.6
0.4
0.2
0.0
0 1 2 3 4 5 6 7 8 9 10 11 12 13 14 15 16 17 18 19 20 21 22 23 24
Time (hrs)
pSB401
DMSO[0.1]
DMSO[0.5]
DMSO[1]
DMSO[2]
DMSO[3]
DMSO[4]
DMSO[5]
RC[H]0.1
RC[H]0.5
RC[H]1
RC[H]2
RC[H]3
RC[H]4
RC[H]5

(3A)

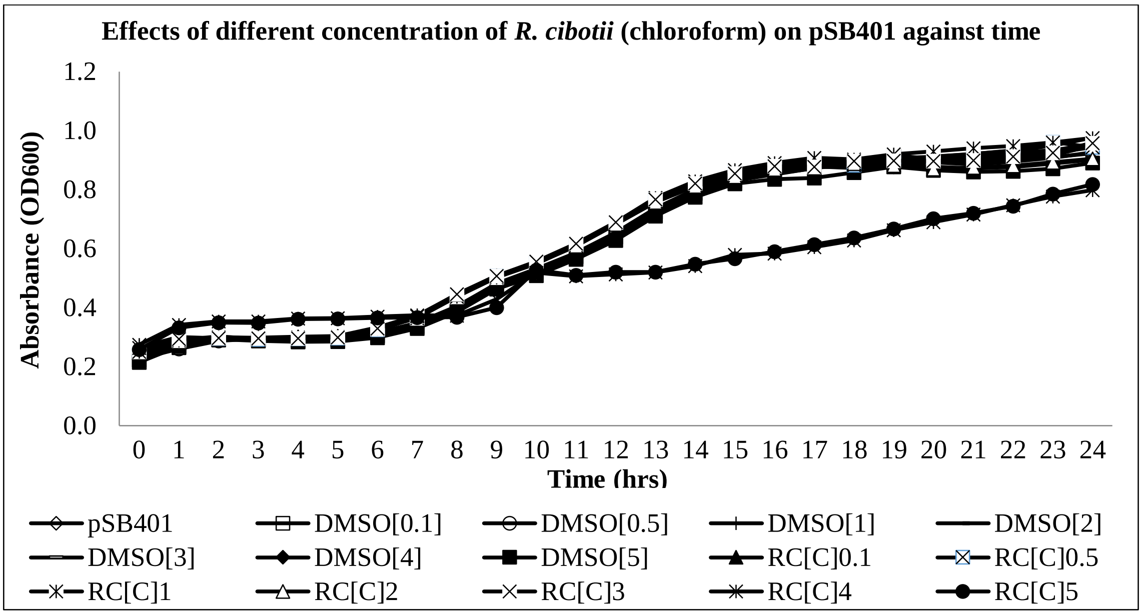
Effects of different concentration of R. cibotii (chloroform) on pSB401 against time
Absorbance (OD600)
1.2
1.0
0.8
0.6
0.4
0.2
0.0
0 1 2 3 4 5 6 7 8 9 10 11 12 13 14 15 16 17 18 19 20 21 22 23 24
Time (hrs)
pSB401
DMSO[0.1]
DMSO[0.5]
DMSO[1]
DMSO[2]
DMSO[3]
DMSO[4]
DMSO[5]
RC[C]0.1
RC[C]0.5
RC[C]1
RC[C]2
RC[C]3
RC[C]4
RC[C]5

(3B)

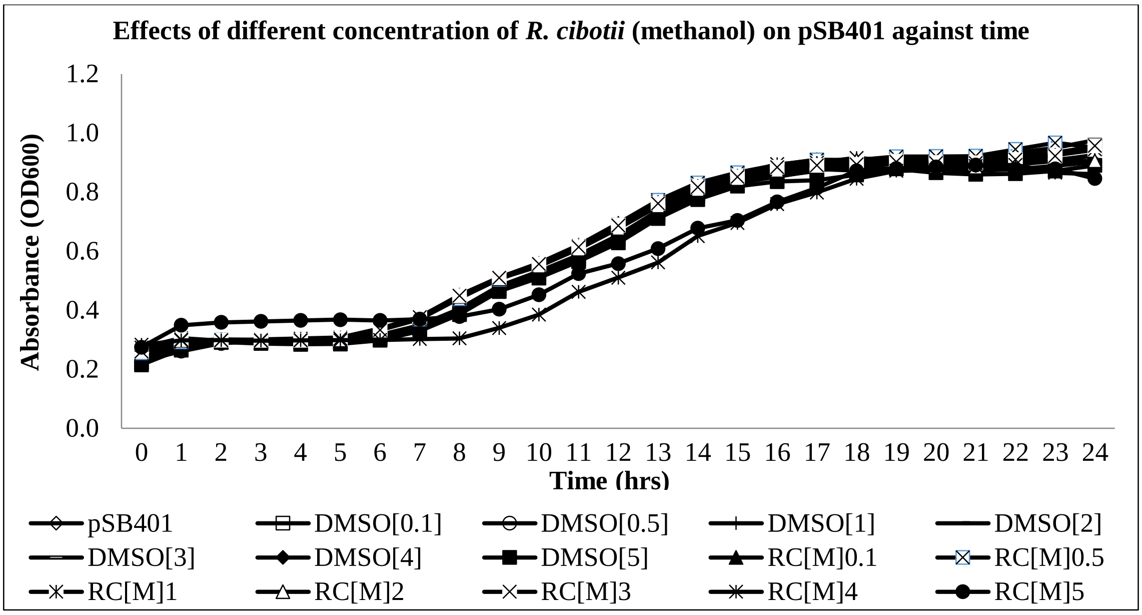
Effects of different concentration of R. cibotii (methanol) on pSB401 against time
Absorbance (OD600)
1.2
1.0
0.8
0.6
0.4
0.2
0.0
0 1 2 3 4 5 6 7 8 9 10 11 12 13 14 15 16 17 18 19 20 21 22 23 24
Time (hrs)
pSB401
DMSO[0.1]
DMSO[0.5]
DMSO[1]
DMSO[2]
DMSO[3]
DMSO[4]
DMSO[5]
RC[M]0.1
RC[M]0.5
RC[M]1
RC[M]2
RC[M]3
RC[M]4
RC[M]5

(3C)

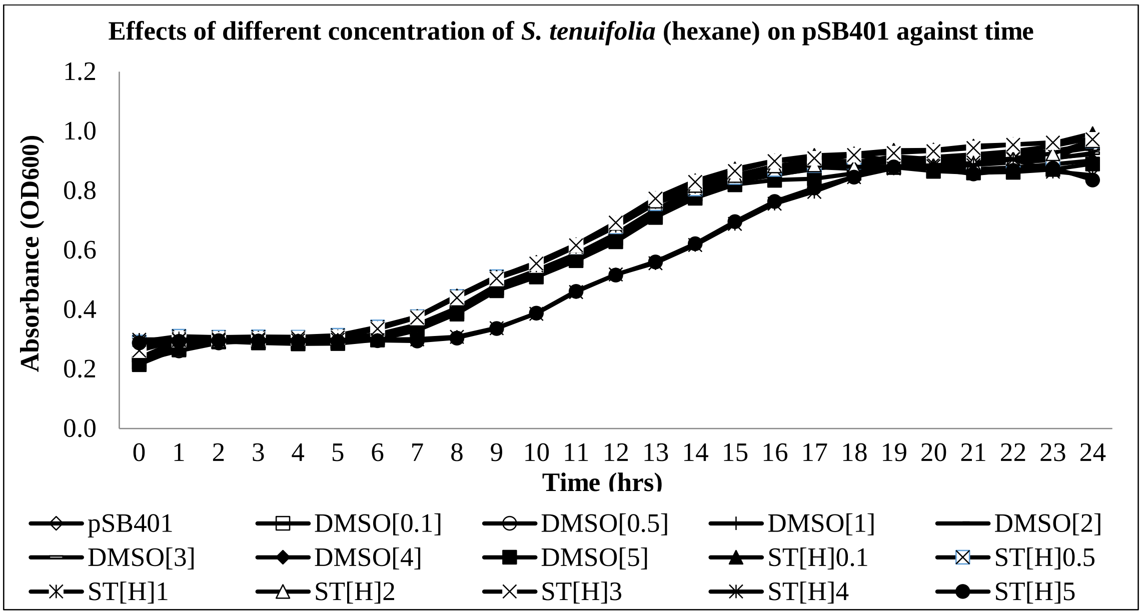
Effects of different concentration of S. tenuifolia (hexane) on pSB401 against time
Absorbance (OD600)
1.2
1.0
0.8
0.6
0.4
0.2
0.0
0 1 2 3 4 5 6 7 8 9 10 11 12 13 14 15 16 17 18 19 20 21 22 23 24
Time (hrs)
pSB401
DMSO[0.1]
DMSO[0.5]
DMSO[1]
DMSO[2]
DMSO[3]
DMSO[4]
DMSO[5]
ST[H]0.1
ST[H]0.5
ST[H]1
ST[H]2
ST[H]3
ST[H]4
ST[H]5

(4A)

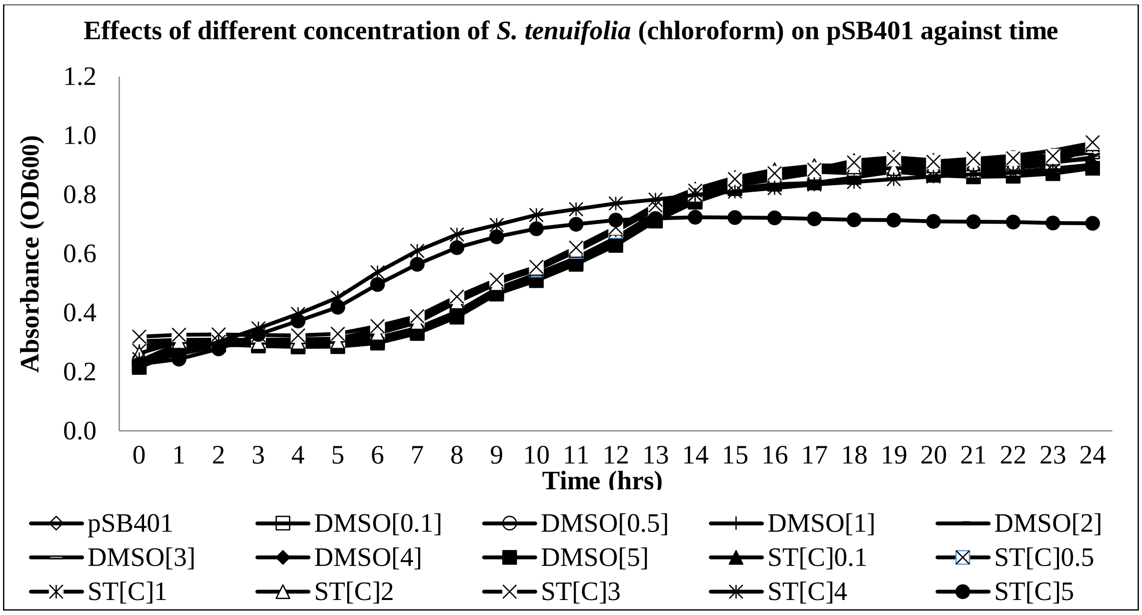

(4B)

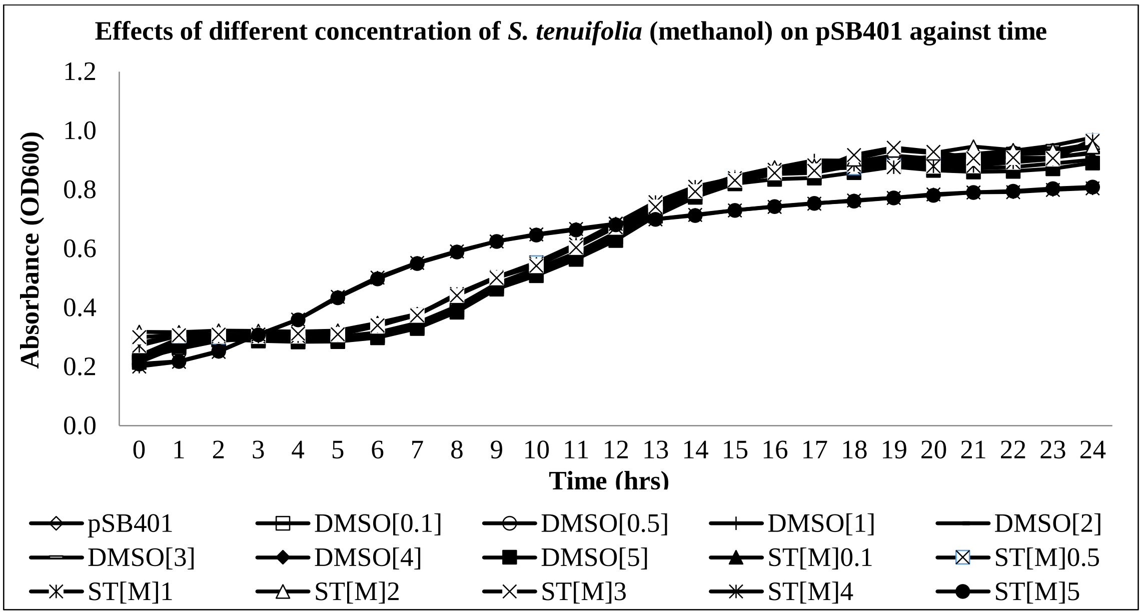

(4C)

Supplementary Figure 2. The growth curve of *E. coli* [pSB401] at the concentration of 0.1, 0.5, 1, 2, 3, 4 and 5 mg/mL of plant extracts in hexane(A), chloroform (B) and methanol(C). (1) *R. pini* (2) *A. dahurica* (3) *R. cibotii* (4) *S. tenuifolia*. The curve for *E. coli* [pSB401] refers to untreated culture while DMSO served as negative control.

(iii) *E. coli* [pSB1075]

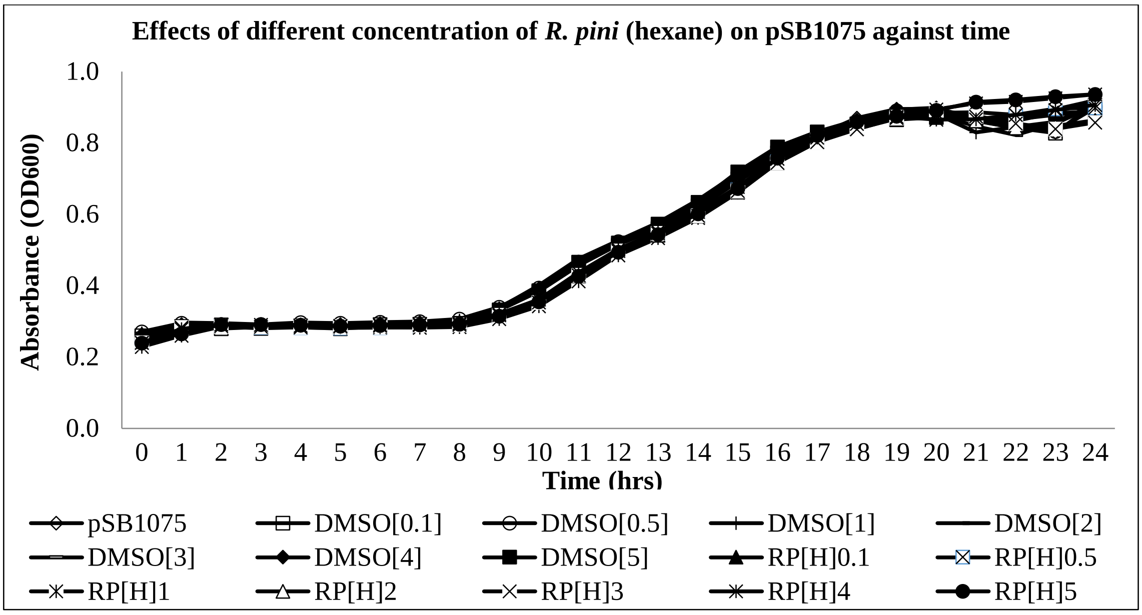

(1A)

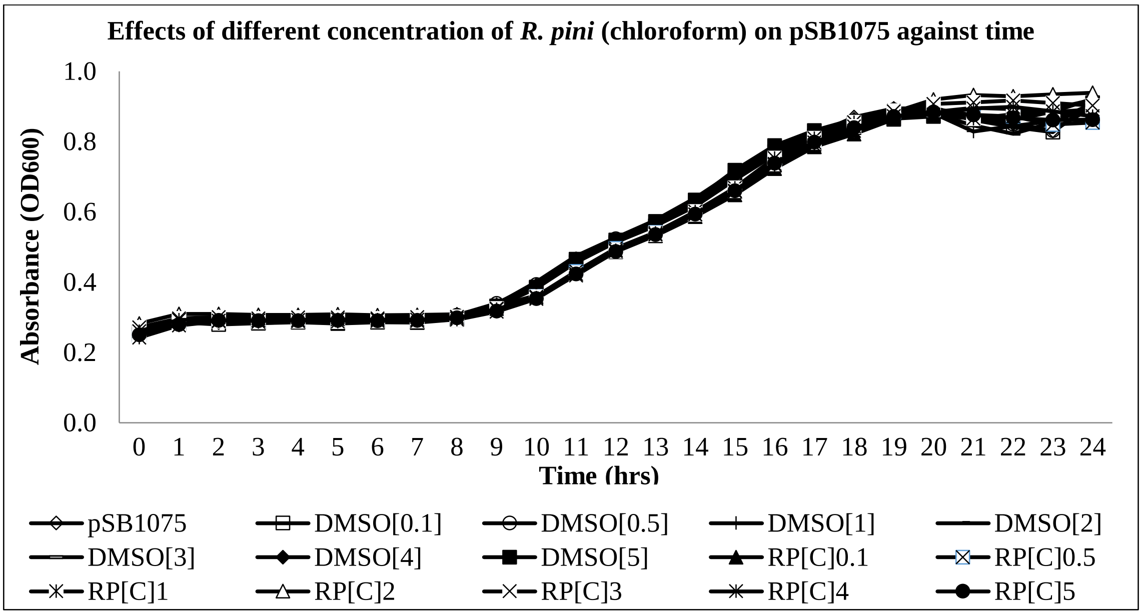

(1B)

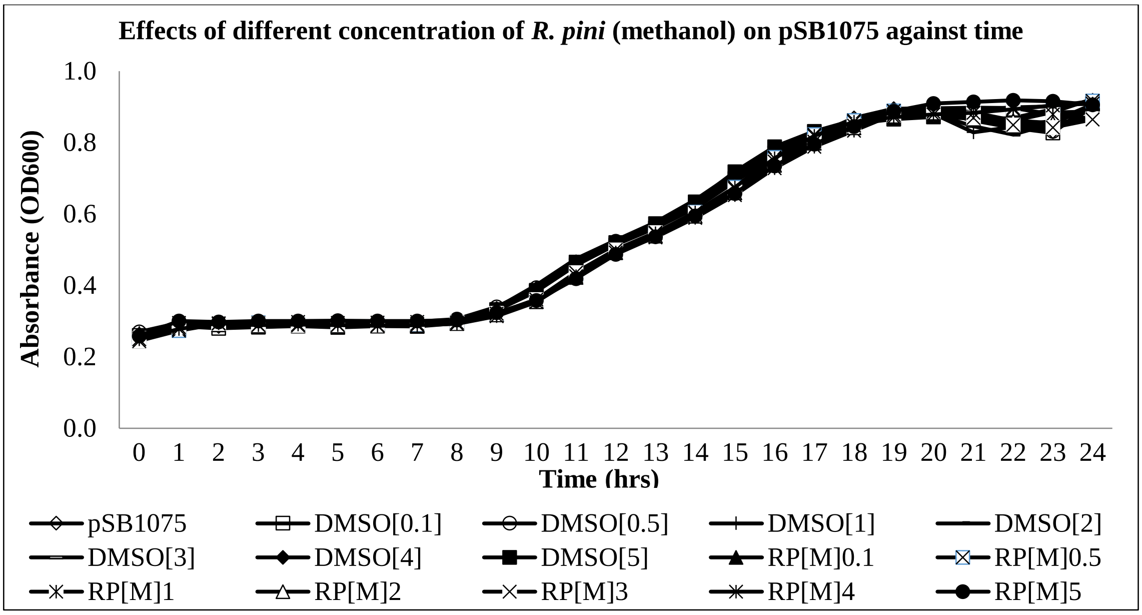

Effects of different concentration of R. pini (methanol) on pSB1075 against time
Absorbance (OD600)
1.0
0.8
0.6
0.4
0.2
0.0
0 1 2 3 4 5 6 7 8 9 10 11 12 13 14 15 16 17 18 19 20 21 22 23 24
Time (hrs)
pSB1075
DMSO[0.1]
DMSO[0.5]
DMSO[1]
DMSO[2]
DMSO[3]
DMSO[4]
DMSO[5]
RP[M]0.1
RP[M]0.5
RP[M]1
RP[M]2
RP[M]3
RP[M]4
RP[M]5

(1C)

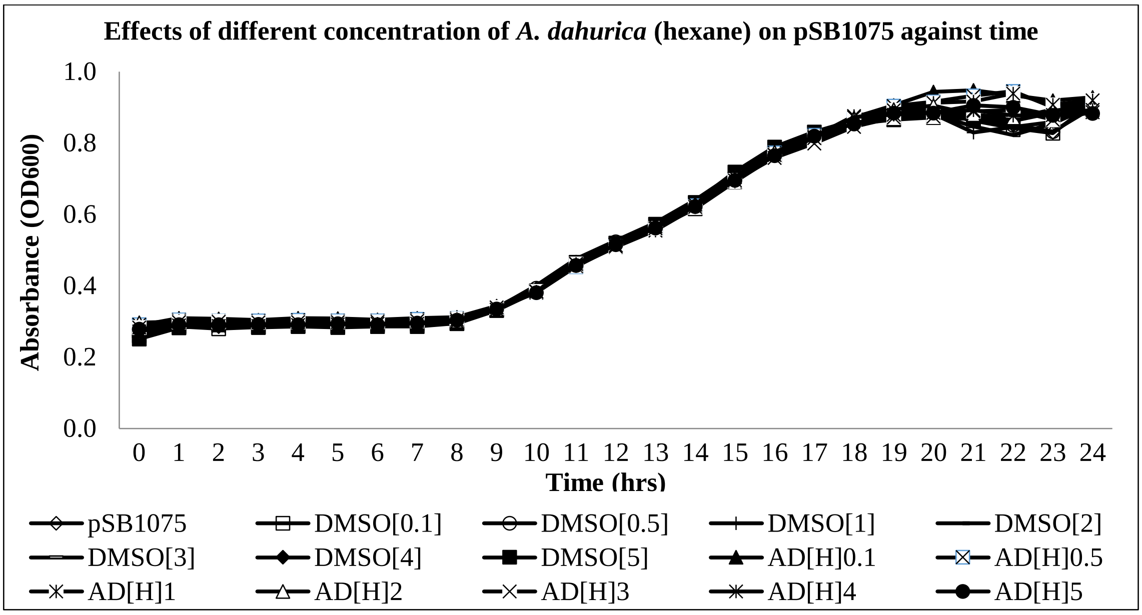

Effects of different concentration of A. dahurica (hexane) on pSB1075 against time
Absorbance (OD600)
1.0
0.8
0.6
0.4
0.2
0.0
0 1 2 3 4 5 6 7 8 9 10 11 12 13 14 15 16 17 18 19 20 21 22 23 24
Time (hrs)
pSB1075
DMSO[0.1]
DMSO[0.5]
DMSO[1]
DMSO[2]
DMSO[3]
DMSO[4]
DMSO[5]
AD[H]0.1
AD[H]0.5
AD[H]1
AD[H]2
AD[H]3
AD[H]4
AD[H]5

(2A)

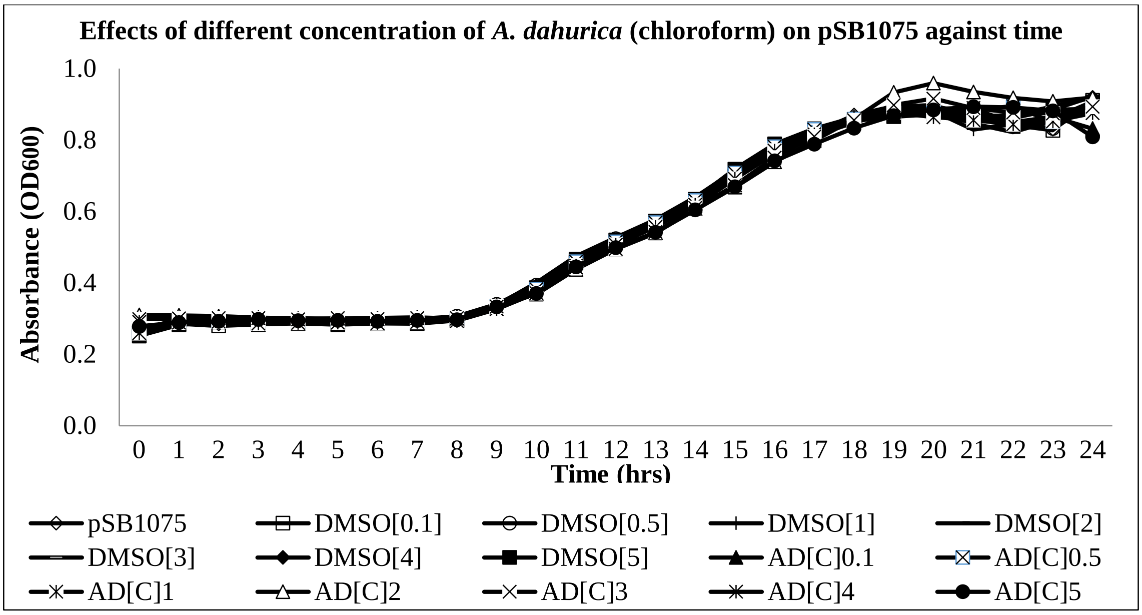
Effects of different concentration of A. dahurica (chloroform) on pSB1075 against time
Absorbance (OD600)
1.0
0.8
0.6
0.4
0.2
0.0
0 1 2 3 4 5 6 7 8 9 10 11 12 13 14 15 16 17 18 19 20 21 22 23 24
Time (hrs)
pSB1075
DMSO[0.1]
DMSO[0.5]
DMSO[1]
DMSO[2]
DMSO[3]
DMSO[4]
DMSO[5]
AD[C]0.1
AD[C]0.5
AD[C]1
AD[C]2
AD[C]3
AD[C]4
AD[C]5

(2B)

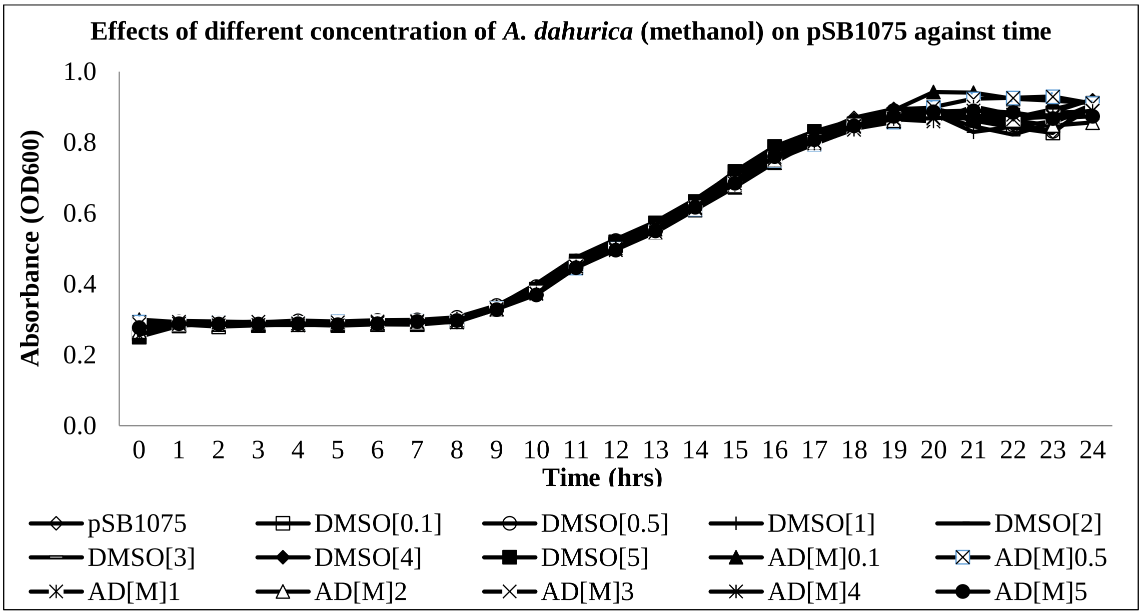
Effects of different concentration of A. dahurica (methanol) on pSB1075 against time
Absorbance (OD600)
1.0
0.8
0.6
0.4
0.2
0.0
0 1 2 3 4 5 6 7 8 9 10 11 12 13 14 15 16 17 18 19 20 21 22 23 24
Time (hrs)
pSB1075
DMSO[0.1]
DMSO[0.5]
DMSO[1]
DMSO[2]
DMSO[3]
DMSO[4]
DMSO[5]
AD[M]0.1
AD[M]0.5
AD[M]1
AD[M]2
AD[M]3
AD[M]4
AD[M]5

(2C)

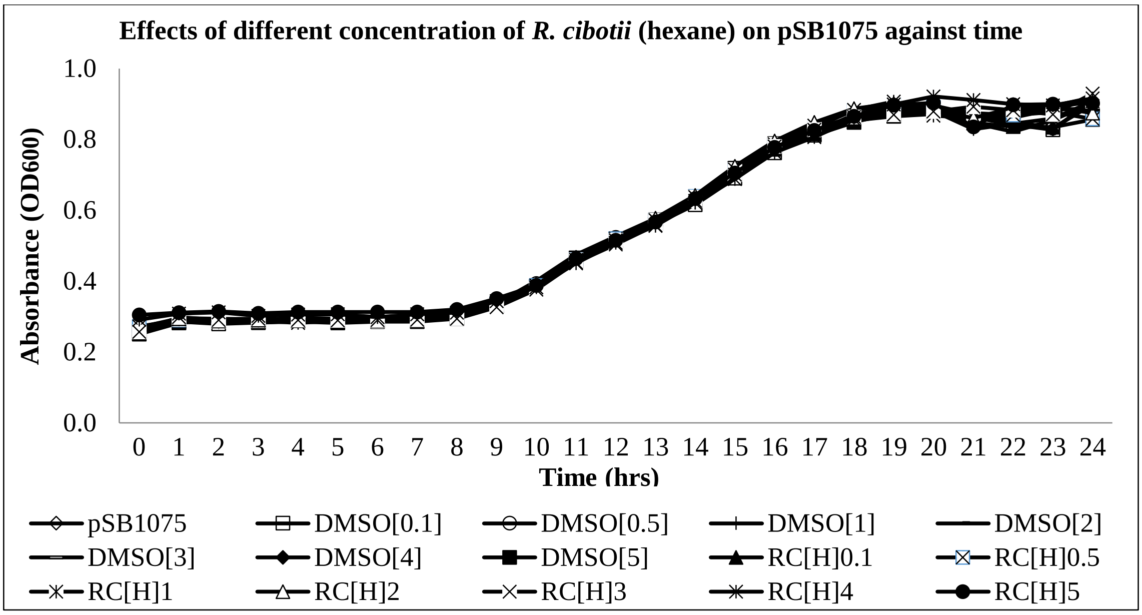

Effects of different concentration of R. cibotii (hexane) on pSB1075 against time
Absorbance (OD600)
1.0
0.8
0.6
0.4
0.2
0.0
0 1 2 3 4 5 6 7 8 9 10 11 12 13 14 15 16 17 18 19 20 21 22 23 24
Time (hrs)
pSB1075
DMSO[0.1]
DMSO[0.5]
DMSO[1]
DMSO[2]
DMSO[3]
DMSO[4]
DMSO[5]
RC[H]0.1
RC[H]0.5
RC[H]1
RC[H]2
RC[H]3
RC[H]4
RC[H]5

(3A)

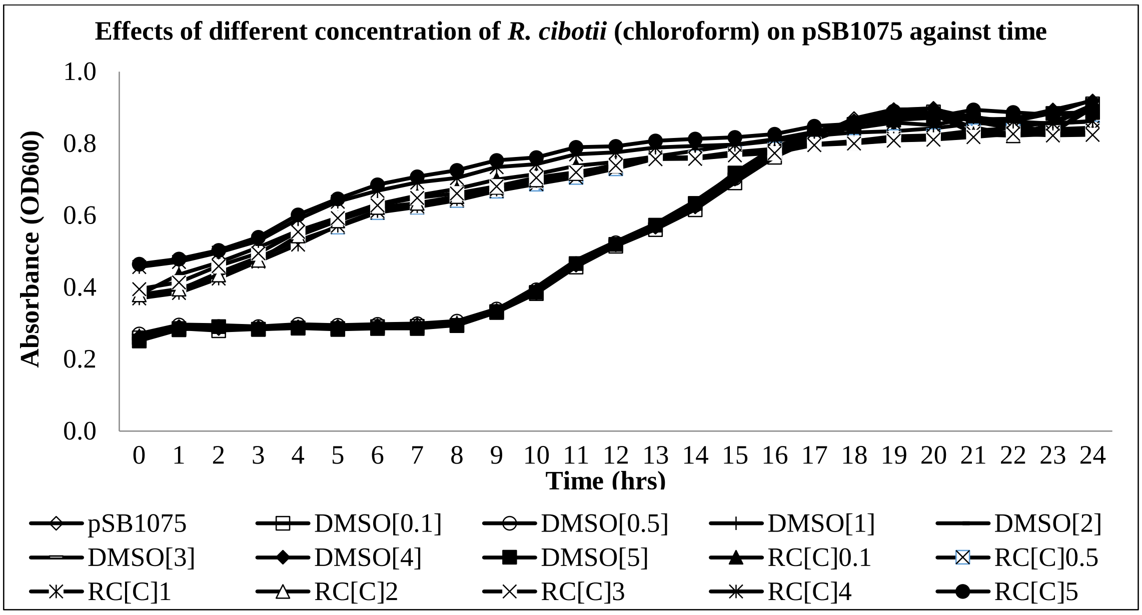

Effects of different concentration of R. cibotii (chloroform) on pSB1075 against time
Absorbance (OD600)
1.0
0.8
0.6
0.4
0.2
0.0
0 1 2 3 4 5 6 7 8 9 10 11 12 13 14 15 16 17 18 19 20 21 22 23 24
Time (hrs)
pSB1075
DMSO[0.1]
DMSO[0.5]
DMSO[1]
DMSO[2]
DMSO[3]
DMSO[4]
DMSO[5]
RC[C]0.1
RC[C]0.5
RC[C]1
RC[C]2
RC[C]3
RC[C]4
RC[C]5

(3B)

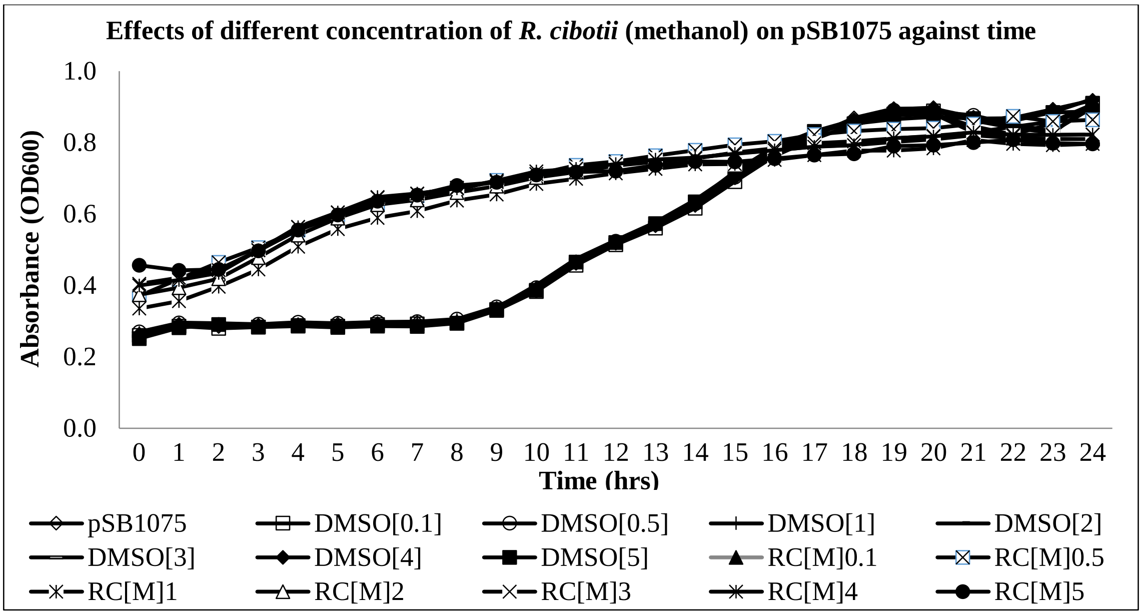
Effects of different concentration of R. cibotii (methanol) on pSB1075 against time
Absorbance (OD600)
1.0
0.8
0.6
0.4
0.2
0.0
0 1 2 3 4 5 6 7 8 9 10 11 12 13 14 15 16 17 18 19 20 21 22 23 24
Time (hrs)
pSB1075
DMSO[0.1]
DMSO[0.5]
DMSO[1]
DMSO[2]
DMSO[3]
DMSO[4]
DMSO[5]
RC[M]0.1
RC[M]0.5
RC[M]1
RC[M]2
RC[M]3
RC[M]4
RC[M]5

(3C)

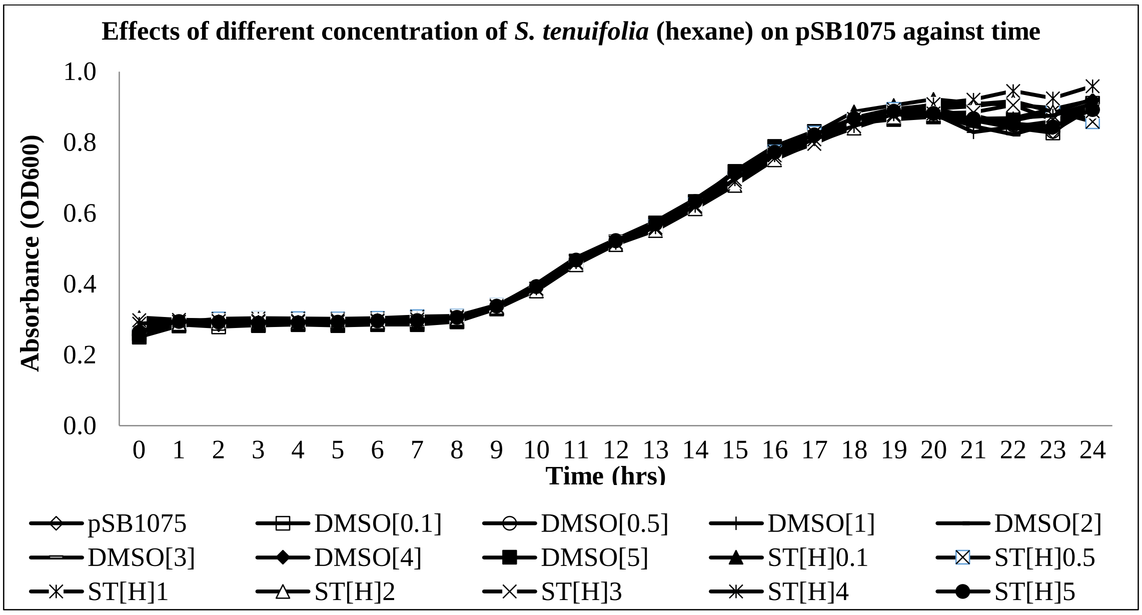
Effects of different concentration of S. tenuifolia (hexane) on pSB1075 against time
Absorbance (OD600)
1.0
0.8
0.6
0.4
0.2
0.0
0 1 2 3 4 5 6 7 8 9 10 11 12 13 14 15 16 17 18 19 20 21 22 23 24
Time (hrs)
pSB1075
DMSO[0.1]
DMSO[0.5]
DMSO[1]
DMSO[2]
DMSO[3]
DMSO[4]
DMSO[5]
ST[H]0.1
ST[H]0.5
ST[H]1
ST[H]2
ST[H]3
ST[H]4
ST[H]5

(4A)

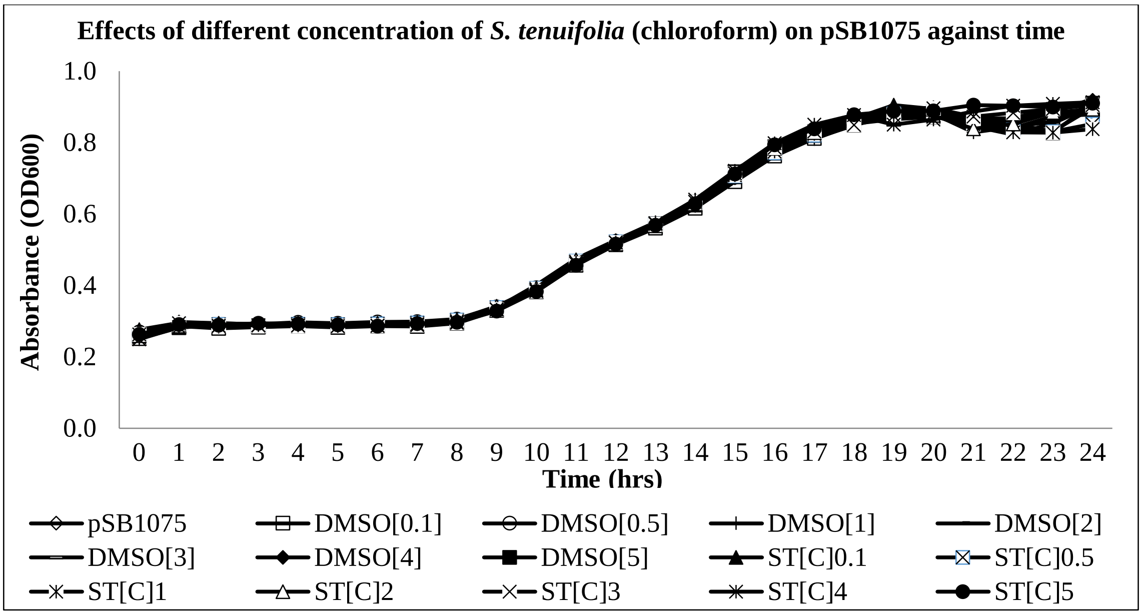

(4B)

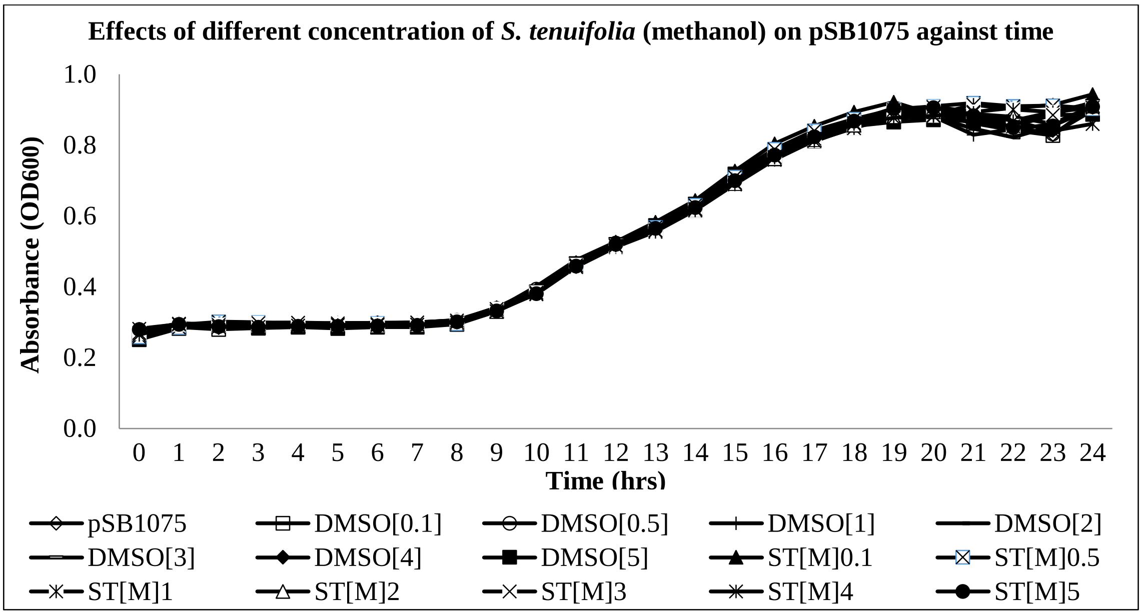

(4C)

Supplementary Figure 3. The growth curve of *E. coli* [pSB1075] at the concentration of 0.1, 0.5, 1, 2, 3, 4 and 5 mg/mL of plant extracts in hexane(A), chloroform (B) and methanol(C). (1) *R. pini* (2) *A. dahurica* (3) *R. cibotii* (4) *S. tenuifolia*. The curve for *E. coli* [pSB1075] refers to untreated culture while DMSO served as negative control.

(iv) *Pseudomonas aeruginosa* PA01

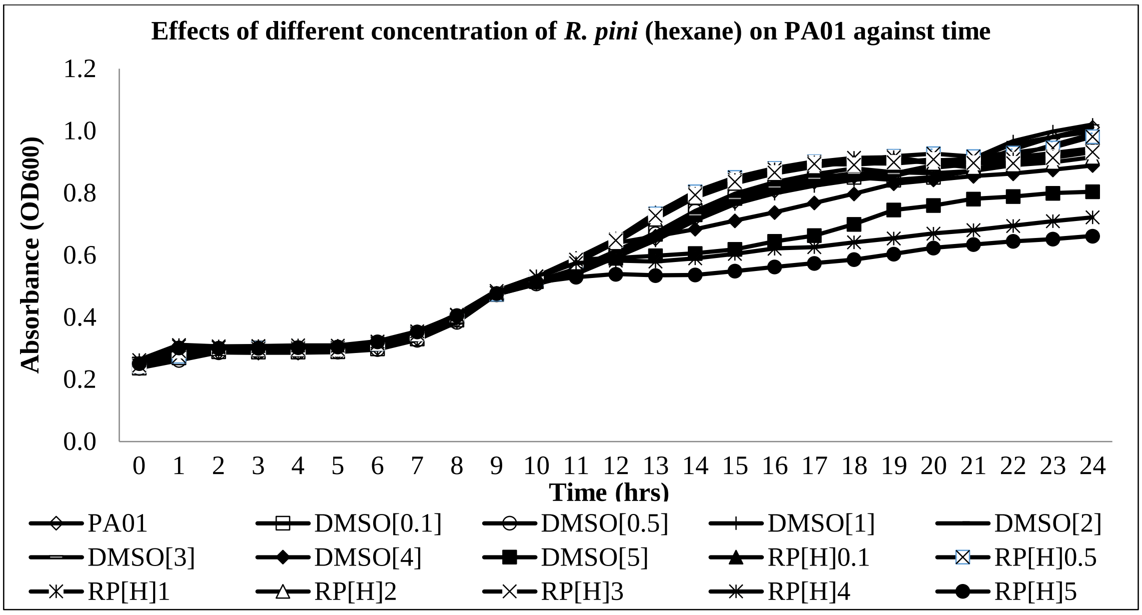

(1A)

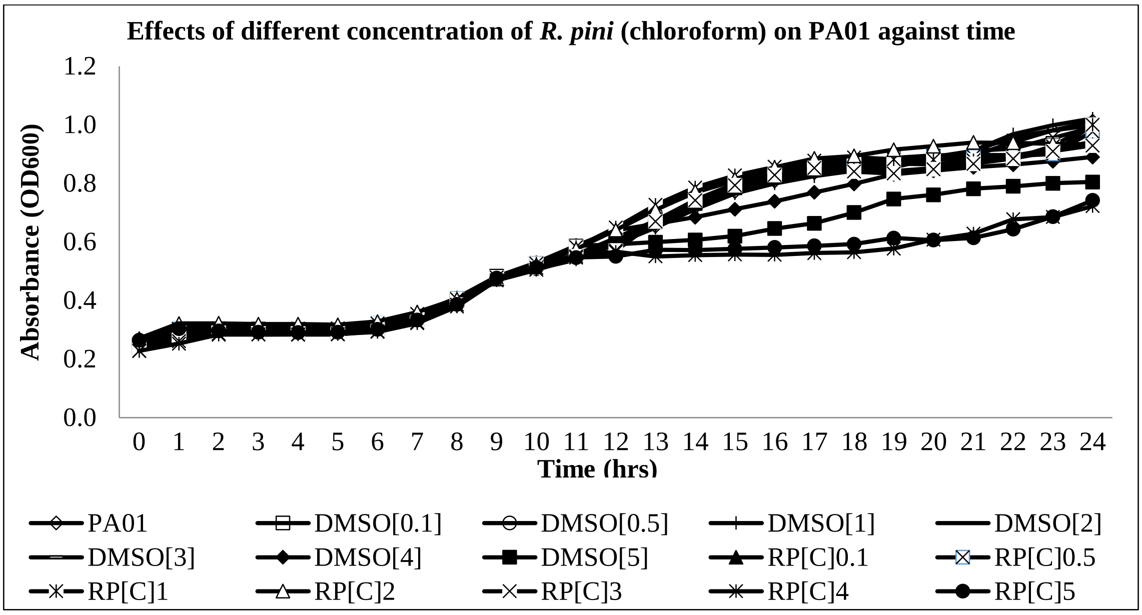

(1B)

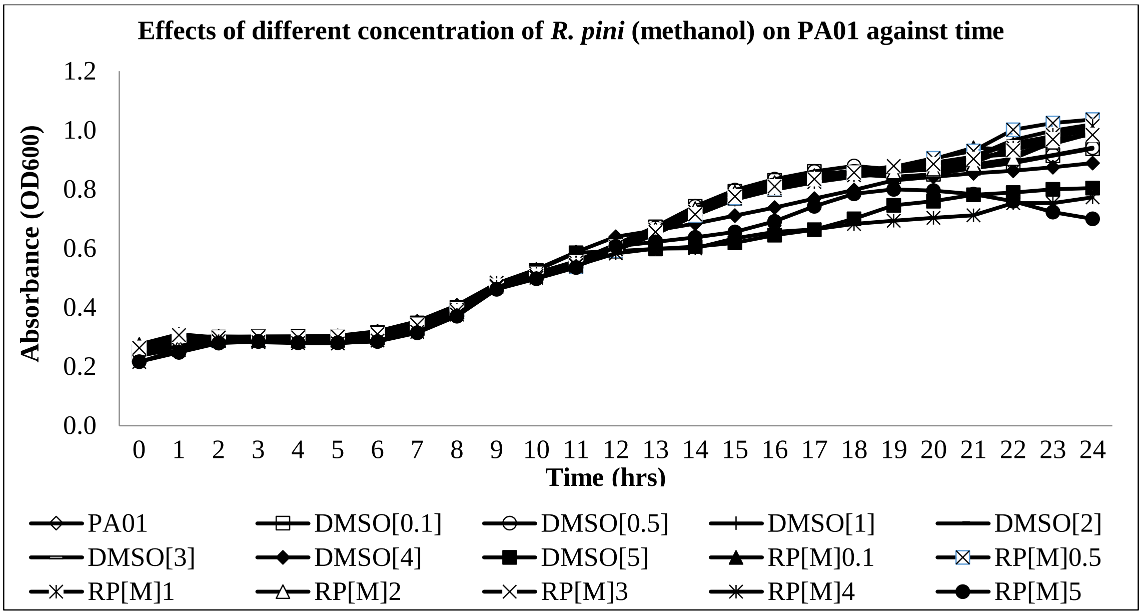
Effects of different concentration of R. pini (methanol) on PA01 against time
Absorbance (OD600)
1.2
1.0
0.8
0.6
0.4
0.2
0.0
0 1 2 3 4 5 6 7 8 9 10 11 12 13 14 15 16 17 18 19 20 21 22 23 24
Time (hrs)
PA01
DMSO[0.1]
DMSO[0.5]
DMSO[1]
DMSO[2]
DMSO[3]
DMSO[4]
DMSO[5]
RP[M]0.1
RP[M]0.5
RP[M]1
RP[M]2
RP[M]3
RP[M]4
RP[M]5

(1C)

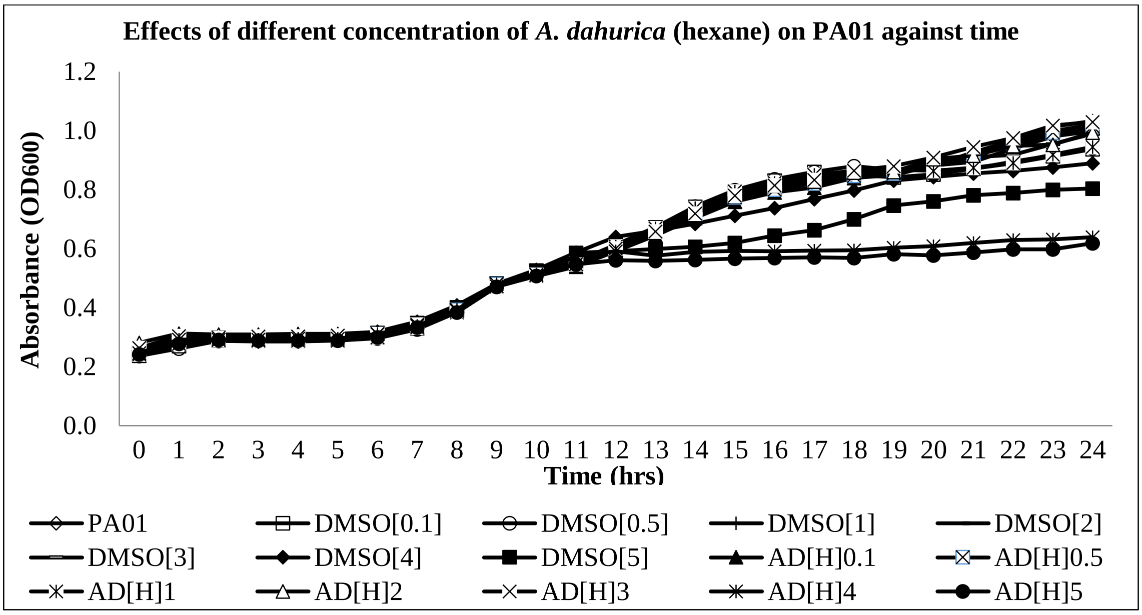
Effects of different concentration of A. dahurica (hexane) on PA01 against time
Absorbance (OD600)
1.2
1.0
0.8
0.6
0.4
0.2
0.0
0 1 2 3 4 5 6 7 8 9 10 11 12 13 14 15 16 17 18 19 20 21 22 23 24
Time (hrs)
PA01
DMSO[0.1]
DMSO[0.5]
DMSO[1]
DMSO[2]
DMSO[3]
DMSO[4]
DMSO[5]
AD[H]0.1
AD[H]0.5
AD[H]1
AD[H]2
AD[H]3
AD[H]4
AD[H]5

(2A)

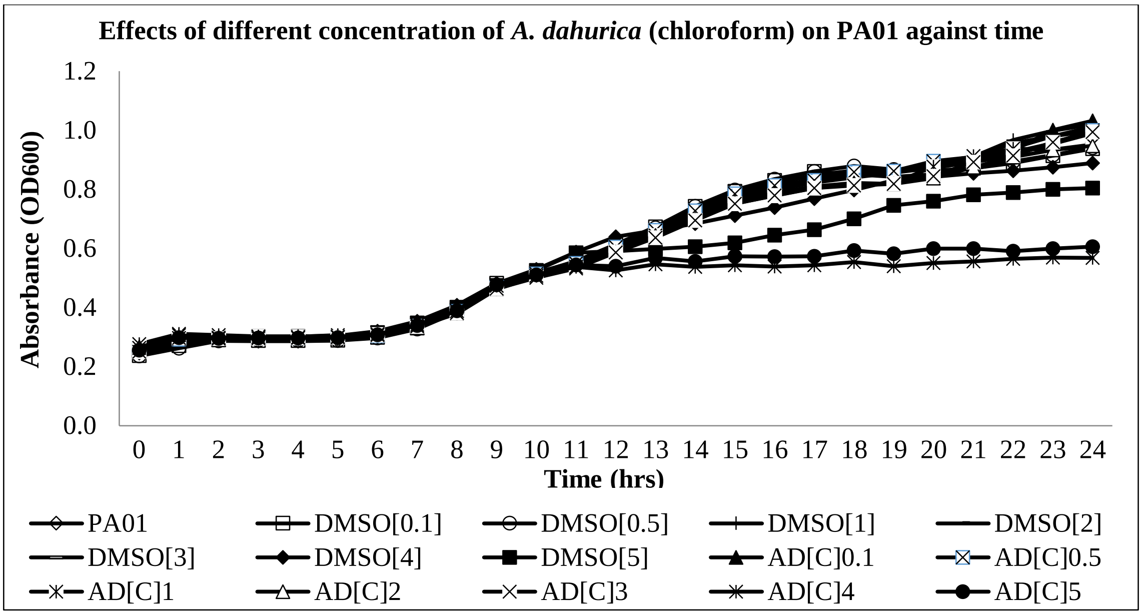
Effects of different concentration of A. dahurica (chloroform) on PA01 against time
Absorbance (OD600)
1.2
1.0
0.8
0.6
0.4
0.2
0.0
0 1 2 3 4 5 6 7 8 9 10 11 12 13 14 15 16 17 18 19 20 21 22 23 24
Time (hrs)
PA01
DMSO[0.1]
DMSO[0.5]
DMSO[1]
DMSO[2]
DMSO[3]
DMSO[4]
DMSO[5]
AD[C]0.1
AD[C]0.5
AD[C]1
AD[C]2
AD[C]3
AD[C]4
AD[C]5

(2B)

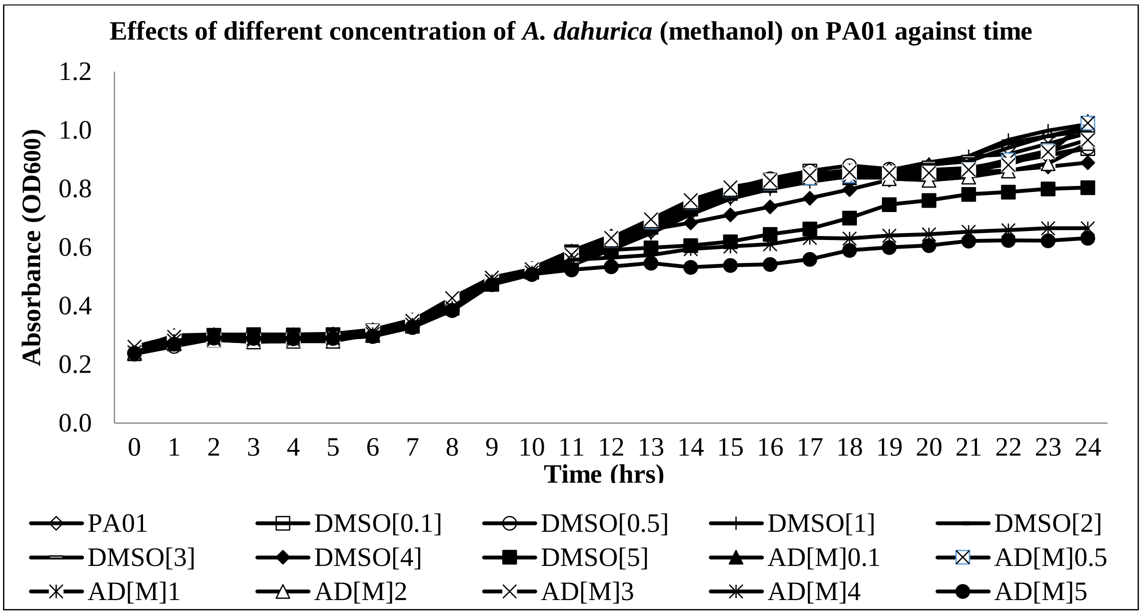
Effects of different concentration of A. dahurica (methanol) on PA01 against time
Absorbance (OD600)
1.2
1.0
0.8
0.6
0.4
0.2
0.0
0 1 2 3 4 5 6 7 8 9 10 11 12 13 14 15 16 17 18 19 20 21 22 23 24
Time (hrs)
PA01
DMSO[0.1]
DMSO[0.5]
DMSO[1]
DMSO[2]
DMSO[3]
DMSO[4]
DMSO[5]
AD[M]0.1
AD[M]0.5
AD[M]1
AD[M]2
AD[M]3
AD[M]4
AD[M]5

(2C)

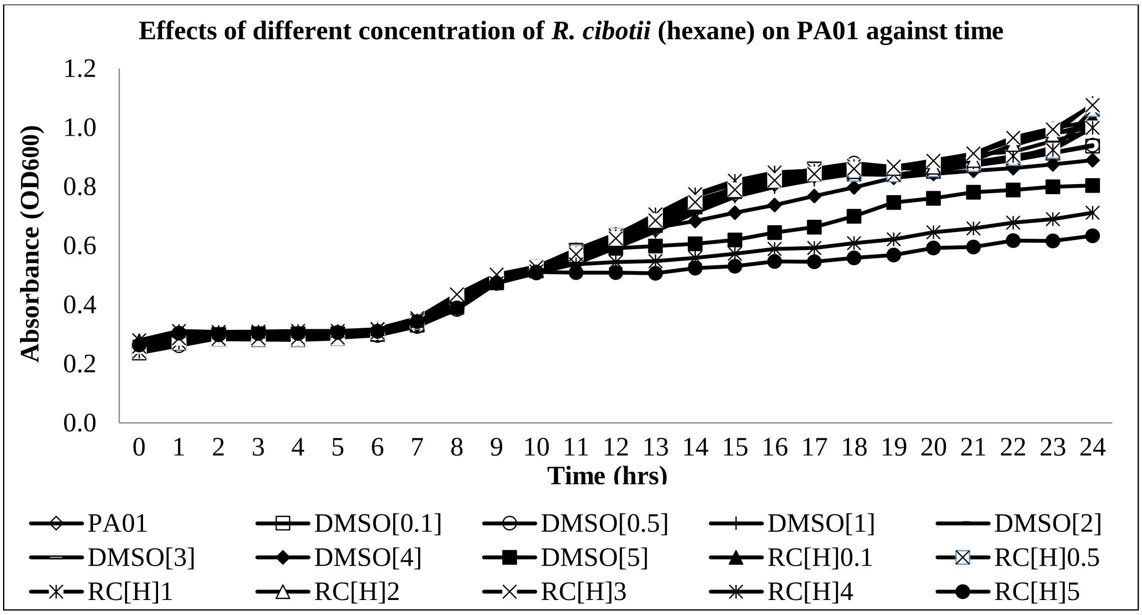
Effects of different concentration of R. cibotii (hexane) on PA01 against time
Absorbance (OD600)
1.2
1.0
0.8
0.6
0.4
0.2
0.0
0 1 2 3 4 5 6 7 8 9 10 11 12 13 14 15 16 17 18 19 20 21 22 23 24
Time (hrs)
PA01
DMSO[0.1]
DMSO[0.5]
DMSO[1]
DMSO[2]
DMSO[3]
DMSO[4]
DMSO[5]
RC[H]0.1
RC[H]0.5
RC[H]1
RC[H]2
RC[H]3
RC[H]4
RC[H]5

(3A)

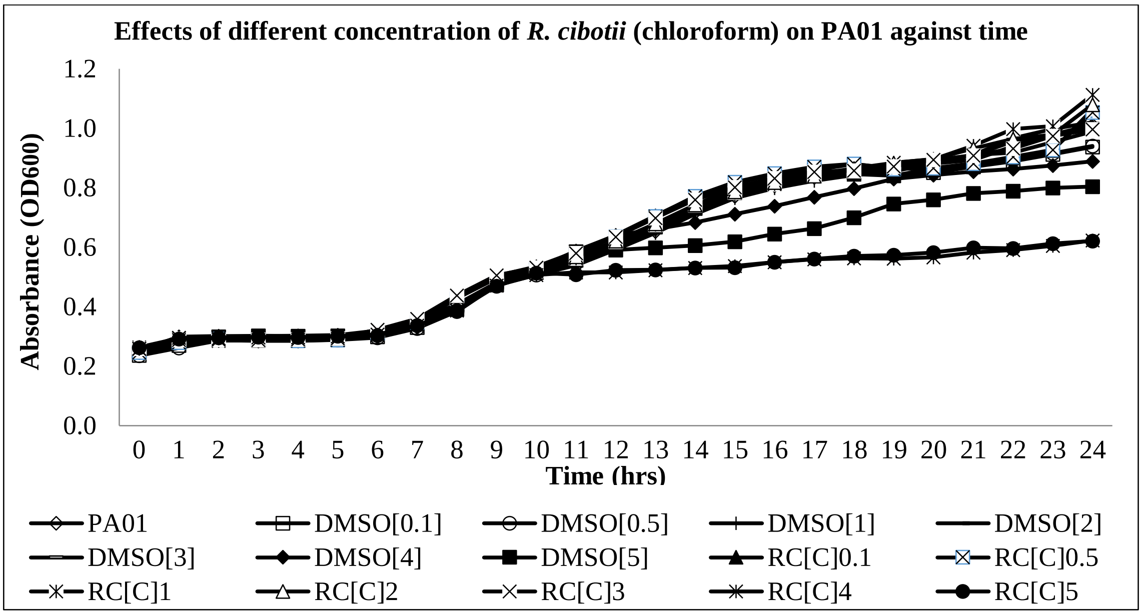
Effects of different concentration of R. cibotii (chloroform) on PA01 against time
Absorbance (OD600)
1.2
1.0
0.8
0.6
0.4
0.2
0.0
0 1 2 3 4 5 6 7 8 9 10 11 12 13 14 15 16 17 18 19 20 21 22 23 24
Time (hrs)
PA01
DMSO[0.1]
DMSO[0.5]
DMSO[1]
DMSO[2]
DMSO[3]
DMSO[4]
DMSO[5]
RC[C]0.1
RC[C]0.5
RC[C]1
RC[C]2
RC[C]3
RC[C]4
RC[C]5

(3B)

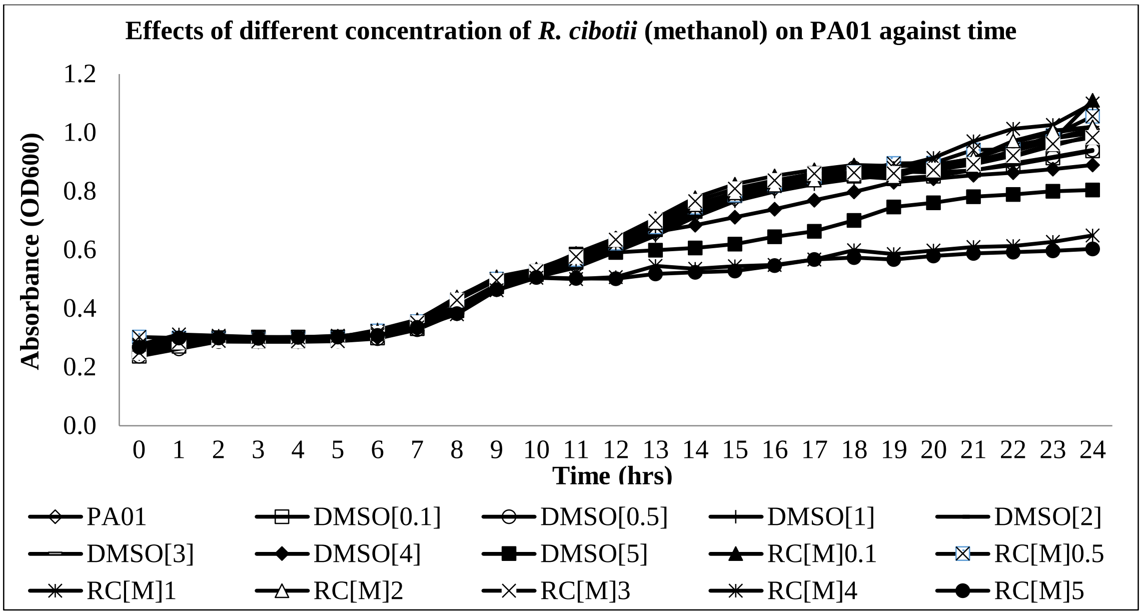
Effects of different concentration of R. cibotii (methanol) on PA01 against time
Absorbance (OD600)
0.0
0.2
0.4
0.6
0.8
1.0
1.2
0 1 2 3 4 5 6 7 8 9 10 11 12 13 14 15 16 17 18 19 20 21 22 23 24
Time (hrs)
PA01
DMSO[0.1]
DMSO[0.5]
DMSO[1]
DMSO[2]
DMSO[3]
DMSO[4]
DMSO[5]
RC[M]0.1
RC[M]0.5
RC[M]1
RC[M]2
RC[M]3
RC[M]4
RC[M]5

(3C)

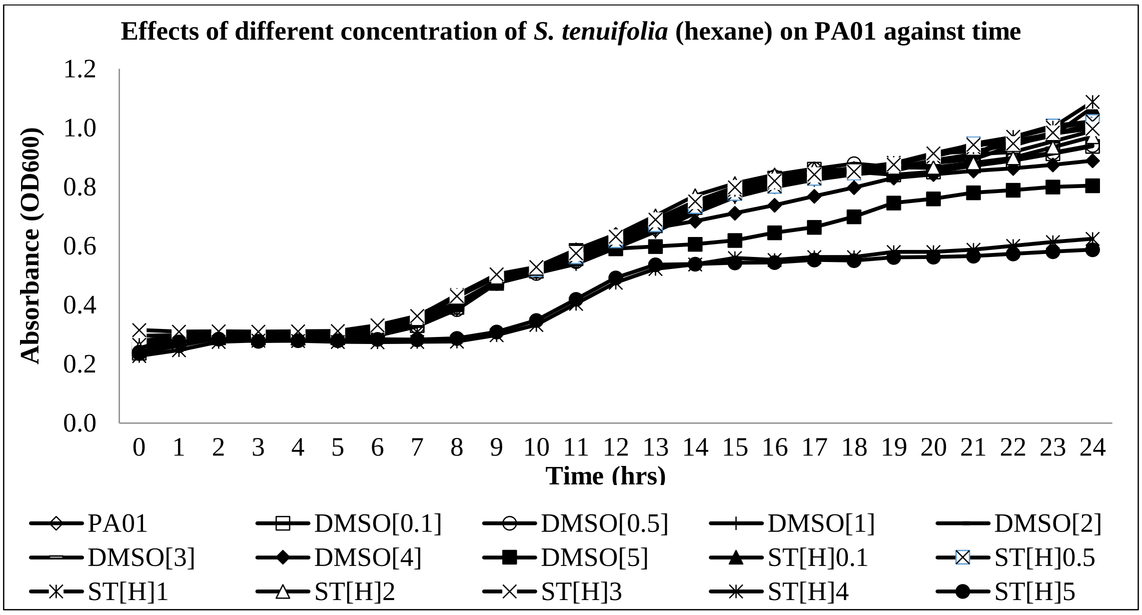
Effects of different concentration of S. tenuifolia (hexane) on PA01 against time
Absorbance (OD600)
0.0
0.2
0.4
0.6
0.8
1.0
1.2
0 1 2 3 4 5 6 7 8 9 10 11 12 13 14 15 16 17 18 19 20 21 22 23 24
Time (hrs)
PA01
DMSO[0.1]
DMSO[0.5]
DMSO[1]
DMSO[2]
DMSO[3]
DMSO[4]
DMSO[5]
ST[H]0.1
ST[H]0.5
ST[H]1
ST[H]2
ST[H]3
ST[H]4
ST[H]5

(4A)

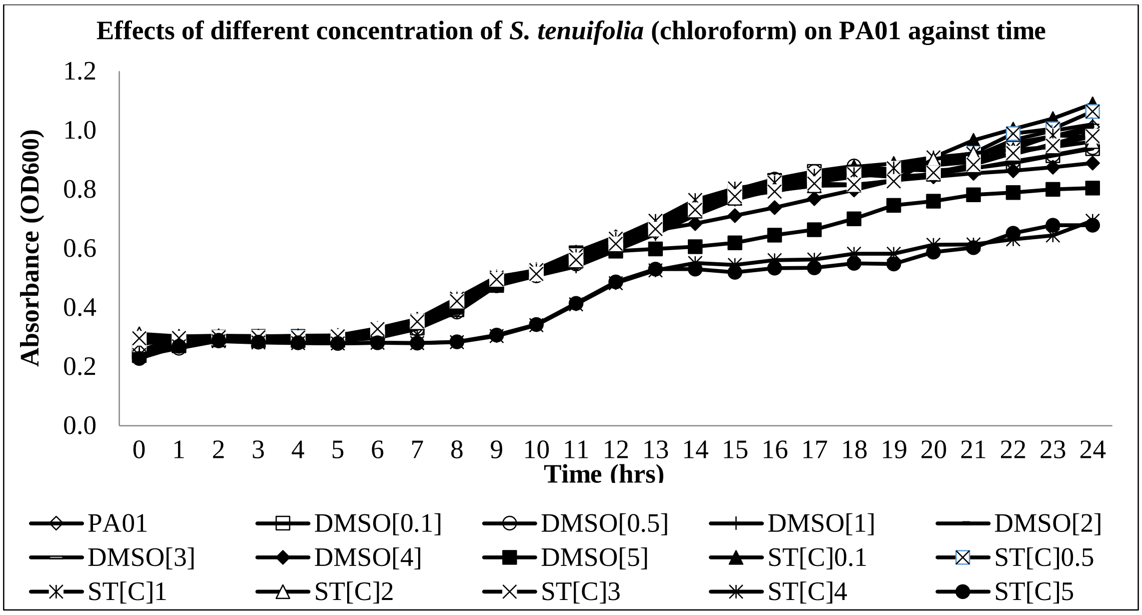

Effects of different concentration of S. tenuifolia (chloroform) on PA01 against time
Absorbance (OD600)
0.0
0.2
0.4
0.6
0.8
1.0
1.2
0 1 2 3 4 5 6 7 8 9 10 11 12 13 14 15 16 17 18 19 20 21 22 23 24
Time (hrs)
PA01
DMSO[0.1]
DMSO[0.5]
DMSO[1]
DMSO[2]
DMSO[3]
DMSO[4]
DMSO[5]
ST[C]0.1
ST[C]0.5
ST[C]1
ST[C]2
ST[C]3
ST[C]4
ST[C]5

(4B)

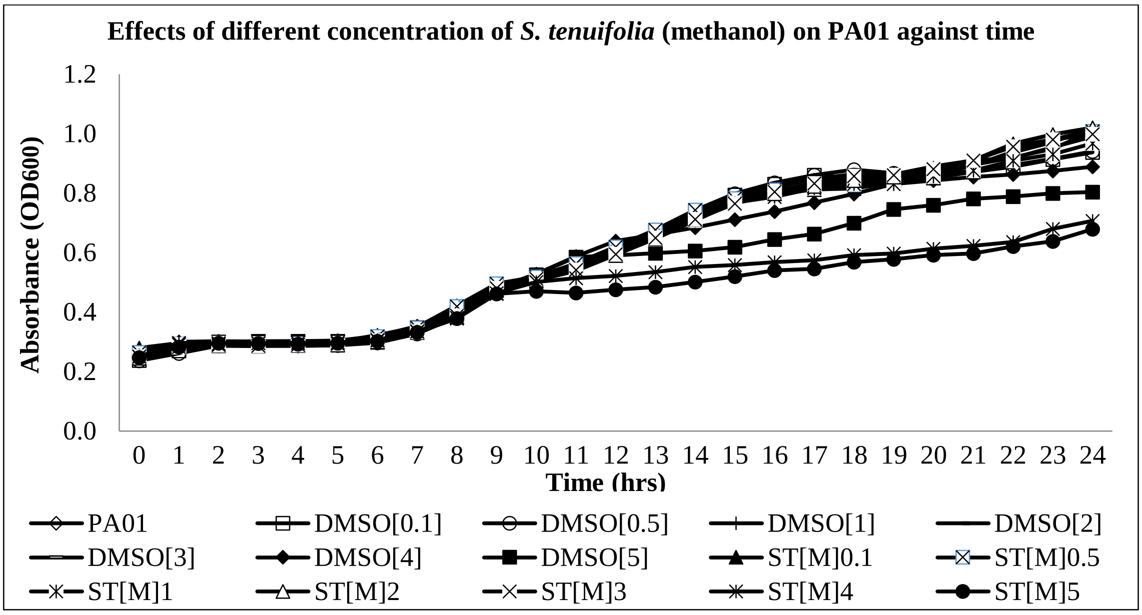

Effects of different concentration of S. tenuifolia (methanol) on PA01 against time
Absorbance (OD600)
0.0
0.2
0.4
0.6
0.8
1.0
1.2
0 1 2 3 4 5 6 7 8 9 10 11 12 13 14 15 16 17 18 19 20 21 22 23 24
Time (hrs)
PA01
DMSO[0.1]
DMSO[0.5]
DMSO[1]
DMSO[2]
DMSO[3]
DMSO[4]
DMSO[5]
ST[M]0.1
ST[M]0.5
ST[M]1
ST[M]2
ST[M]3
ST[M]4
ST[M]5

(4C)

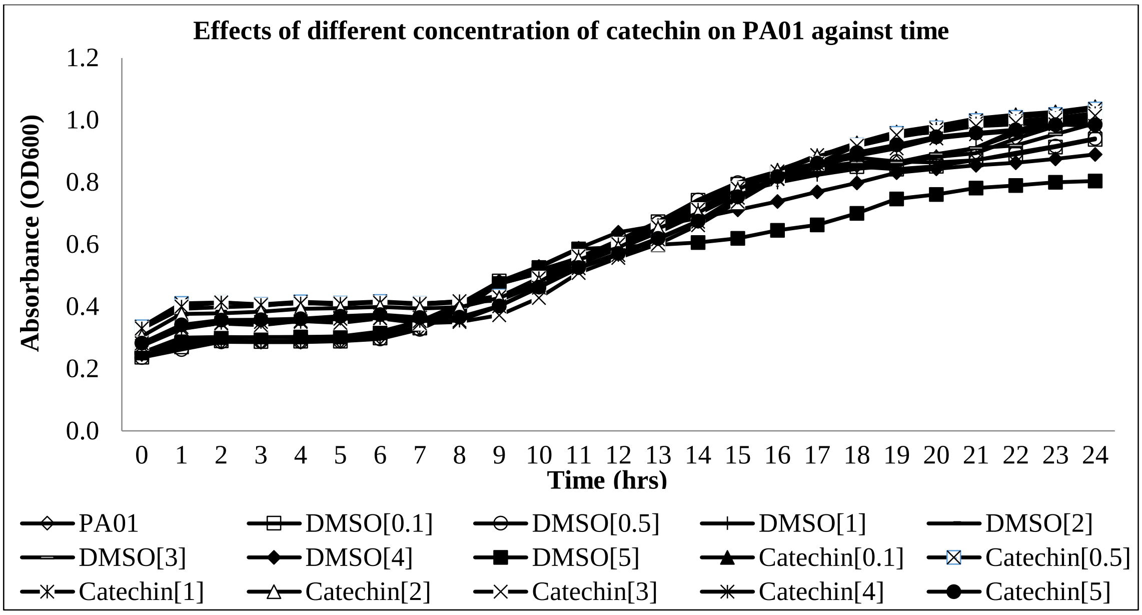

Supplementary Figure 4. The growth curve of *P. aeruginosa* PA01 at the concentration of 0.1, 0.5, 1, 2, 3, 4 and 5 mg/mL of plant extracts in hexane(A), chloroform (B) and methanol(C). (1) *R. pini* (2) *A. dahurica* (3) *R. cibotii* (4) *S. tenuifolia*. The curve for *P. aeruginosa* PAO1 refers to untreated culture while DMSO and catechin served as negative and positive controls, respectively.

B. Swarming Motility Assay

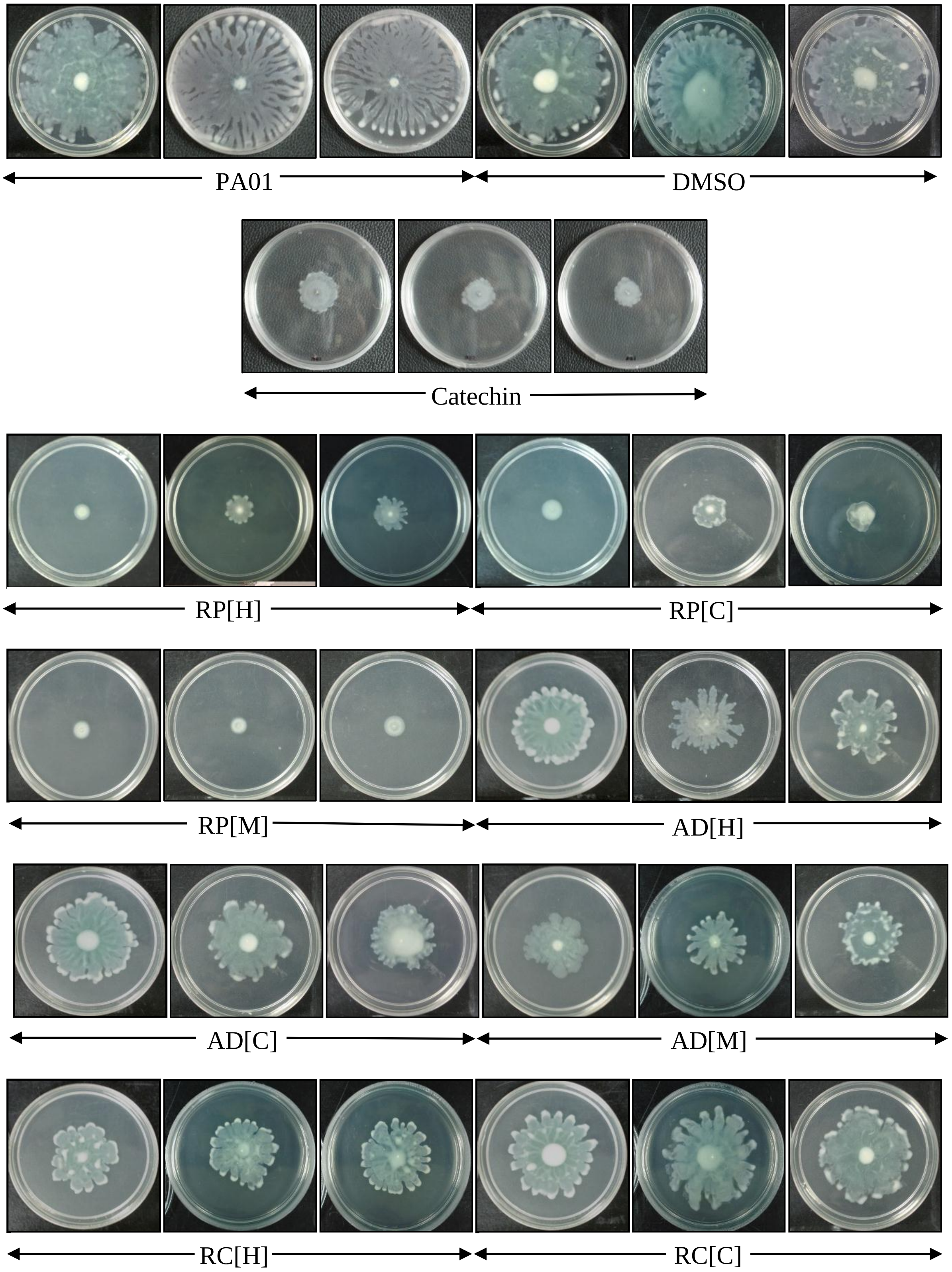

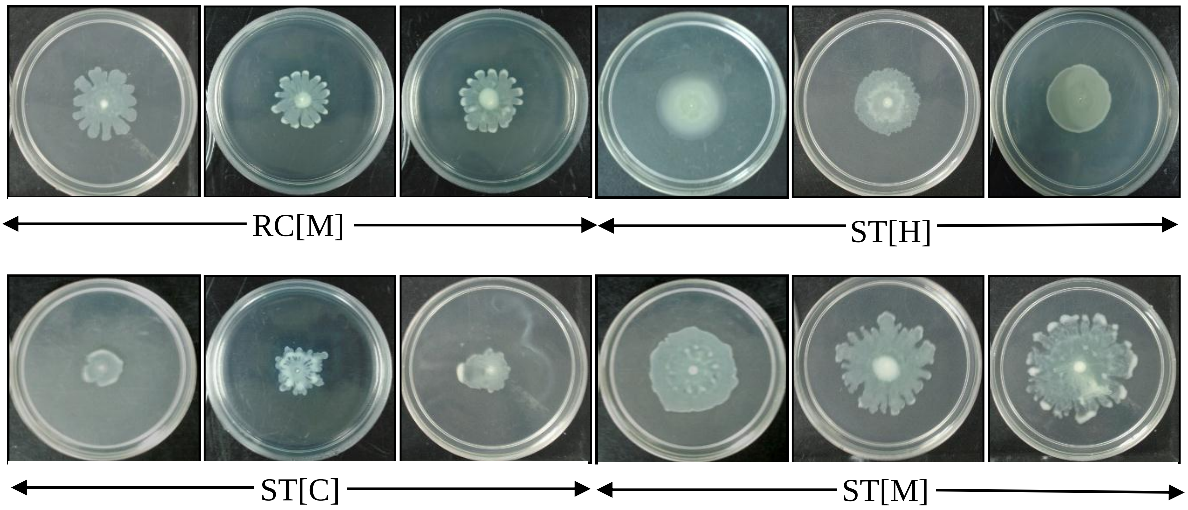

Supplementary Figure 5. The effects of the four plant extracts (*R. pini, A. dahurica, R. cibotii* and *S. tenuifolia*) on the swarming motility in *P. aeruginosa* PAO1 at the concentration of 1 mg/mL in hexane[H], chloroform[C] and methanol[M] extraction. Swarming agar inoculated with *P. aeruginosa* PAO1 alone, *P. aeruginosa* PAO1 supplemented with DMSO (10% v/v) as negative control and *P. aeruginosa* PAO1 supplemented with catechin at 1 mg/mL as positive control.
